# FBXO38 Ubiquitin Ligase Controls Centromere Integrity via ZXDA/B Stability

**DOI:** 10.3389/fcell.2022.929288

**Published:** 2022-06-23

**Authors:** Nikol Dibus, Vladimir Korinek, Lukas Cermak

**Affiliations:** ^1^ Laboratory of Cancer Biology, Institute of Molecular Genetics of the Czech Academy of Sciences, Vestec, Czechia; ^2^ Laboratory of Cell and Developmental Biology, Institute of Molecular Genetics of the Czech Academy of Sciences, Prague, Czechia

**Keywords:** proteasome, ubiquitin ligase, protein degradation, cullin, zinc finger protein, centromere

## Abstract

Alterations in the gene encoding the E3 ubiquitin ligase substrate receptor FBXO38 have been associated with several diseases, including early-onset motor neuronopathy. However, the cellular processes affected by the enzymatic action of FBXO38 are not yet known. Here, we identify the zinc finger proteins ZXDA/B as its interaction partners. FBXO38 controls the stability of ZXDA/B proteins via ubiquitination and proteasome-dependent degradation. We show that ZXDA/B proteins associate with the centromeric protein CENP-B and that the interaction between ZXDA/B and FBXO38 or CENP-B is mutually exclusive. Functionally, ZXDA/B factors control the protein level of chromatin-associated CENP-B. Furthermore, their inappropriate stabilization leads to upregulation of CENP-A and CENP-B positive centromeric chromatin. Thus we demonstrate a previously unknown role of cullin-dependent protein degradation in the control of centromeric chromatin integrity.

## Introduction

The ubiquitin-proteasome pathway is a cellular system that shapes the temporal and spatial proteome via targeted protein degradation. This control is achieved via the specific recognition of targeted proteins by ubiquitin ligases (Senft et al., 2018). Multi-subunit E3 ubiquitin ligases allow system versatility by using separate modules for substrate recognition and ubiquitination (Skaar et al., 2014). F-box containing protein 38 (FBXO38; also known as MoKA) is a substrate receptor for SKP1-CUL1-dependent ubiquitin ligase (SCF). Initially, it was discovered as a modulator of transcription factor Krüppel-like factor 7 (KLF7) activity (Smaldone et al., 2004). In addition, FBXO38 stability is controlled via ubiquitin-specific peptidase 7 (USP7), and FBXO38 protein expression was shown to be required for proper cytokinesis (Georges et al., 2019). However, as a ubiquitin ligase, SCF^FBXO38^ is still insufficiently characterized. So far, the only potential substrate identified is programmed cell death 1 (PD-1) (Meng et al., 2018).

Significantly, two FBXO38 gene mutations were found in patients with early-onset distal hereditary motor neuronopathy, indicating its role in the homeostasis of the nervous system (Sumner et al., 2013; Akcimen et al., 2019). Interestingly, another mutation was discovered in the study of monozygotic twins with discordant development of gender dysphoria (Morimoto et al., 2017).

Proteins zinc finger X-linked duplicated A and B (ZXDA/B) have no sufficiently described function. Their close homolog and interacting partner ZXDC has been shown to play a role in the transcription control of major histocompatibility complex class II (Al-Kandari et al., 2007). However, there is no significant evidence supporting the role of ZXDA/B in this process.

Centromeres serve as a platform for microtubule attachment during chromosome segregation. They are defined by the tandem arrays of ɑ-satellite DNA repeats and by the epigenetic signature, characterized by substituting histone H3 with its variant centromere protein A (CENP-A) (Earnshaw and Rothfield, 1985; Palmer et al., 1987). CENP-A nucleosomes are assembled during mitotic exit and the G1 phase of the cell cycle (Jansen et al., 2007). CENP-A depletion, once the kinetochore is already tethered to the centromere, does not disrupt chromosome segregation since its fidelity is ensured by the CENP-B DNA binding protein (Hoffmann et al., 2016). CENP-B protein, homologous to transposases, binds DNA motif (CENP-B box) frequently found in centromeric repetitions (Masumoto et al., 1989; Muro et al., 1992). Functionally, it supports the stabilization of the kinetochore structure through direct binding to CENP-A and by maintaining the CENP-C protein at the centromeres (Fachinetti et al., 2015; Fujita et al., 2015). Moreover, studies on human artificial chromosomes show that forming a functional centromere requires CENP-B protein-dependent CENP-A loading (Ohzeki et al., 2002; Okada et al., 2007). Interestingly, CENP-B is missing from the Y chromosome due to the absence of CENP-B boxes (Masumoto et al., 1989) and from ectopically-derived neocentromeres (du Sart et al., 1997). As a possible consequence, the Y chromosome exhibits an increased missegregation rate compared to other chromosomes (Fachinetti et al., 2015). Furthermore, CENP-B controls the epigenetic signature of centromeres by recruiting various histone modifiers, including tethering histone H3.3 chaperone DAXX to pericentric regions in a SUMO2-dependent manner (Okada et al., 2007; Drane et al., 2010; Morozov et al., 2017; Otake et al., 2020). Notably, Cenp-B-deficient mice are viable, but they exhibit decreased body weight, lower sperm count, and age-dependent deterioration of female reproductive organs (Hudson et al., 1998; Kapoor et al., 1998; Perez-Castro et al., 1998; Fowler et al., 2000; Fowler et al., 2004).

Here, we have revealed the previously unknown role of SCF^FBXO38^ ubiquitin ligase. We identified ZXDA/B zinc finger proteins as its substrates. Furthermore, we show that ZXDA/B proteins are responsible for CENP-B protein stabilization at centromeres, a process negatively controlled by FBXO38.

## Results

### FBXO38 Interacts With ZXDA/B in the Nucleus

**FIGURE 1:**
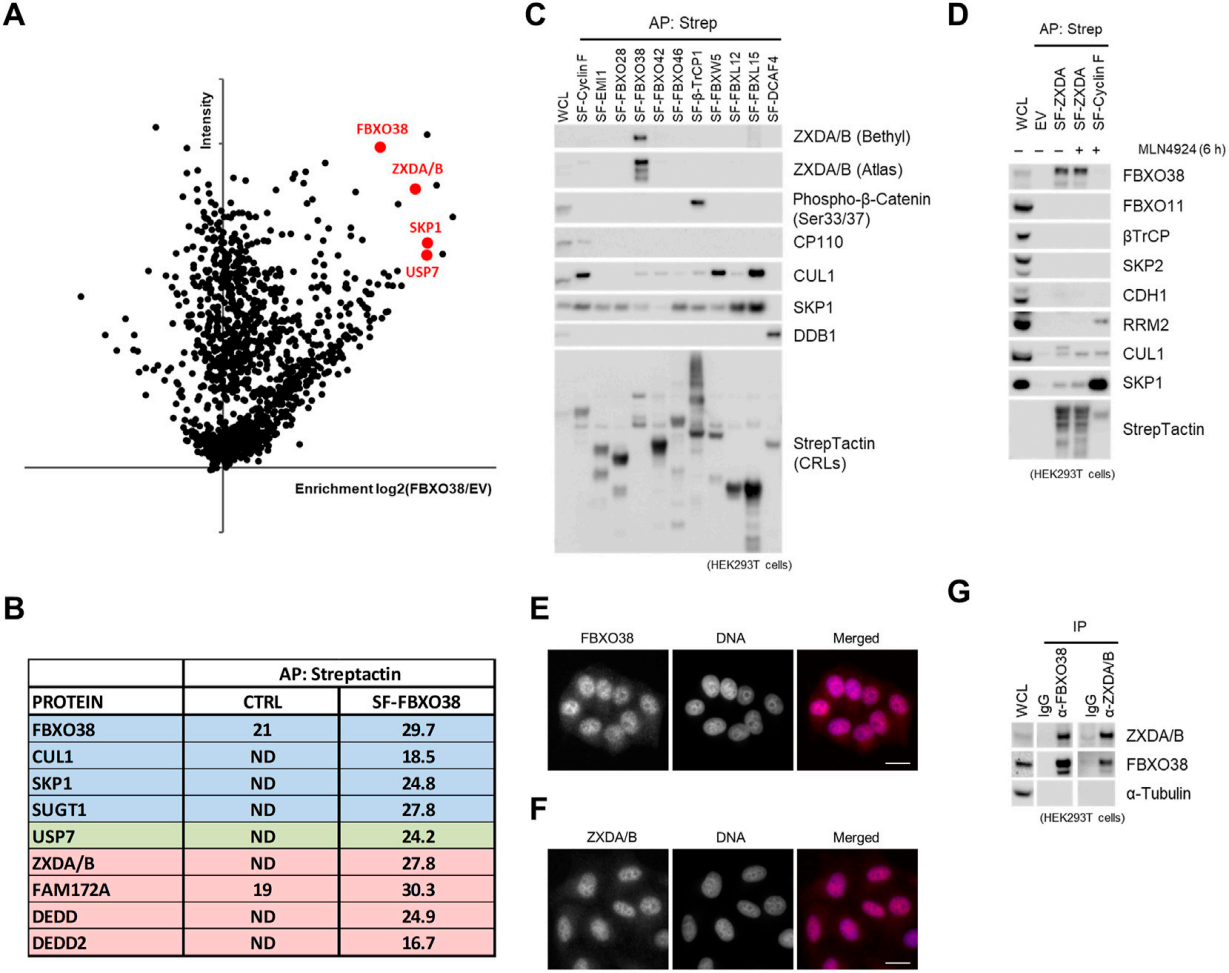
FBXO38 interacts with ZXDA/B in the nucleus. **(A)** Schematic representation of mass-spectrometry identification of FBXO38-interacting proteins. Affinity-purified proteins from HeLa cells expressing StrepII-FLAG-tagged FBXO38 (SF-FBXO38) were identified by Liquid Chromatography with Tandem Mass-Spectrometry (LC-MS/MS). Parental HeLa cells without FBXO38 expression were used as a negative control. Proteins that were significantly enriched in FBXO38 purifications compared to the control are in red. The graph represents maximum intensity and the enrichment calculated from the ratio of intensities of identified proteins. **(B)** The list of specific hits enriched in FBXO38 purifications identified by the LC-MS/MS analysis as in **(A)**. Log_2_ intensities from FBXO38 purifications or the control are shown. Components of SCF^FBXO38^ are depicted in blue, putative substrates in red, and other associated proteins in green. **(C)** HEK293T cells were transfected with SF-tagged Cullin-RING ligase substrate receptors (CRLs). Cells were treated with the MLN4924 inhibitor 6 h prior to collecting. Whole-cell lysates (WCL) were affinity purified (AP) using Strep-Tactin resin and immunoblotted as indicated. As positive controls, phospho-β-catenin is shown to interact with β-TrCP, and CP110 is shown to interact with Cyclin **(F)**. Two anti-ZXDA/B antibodies were used to ensure interaction specificity. **(D)** HEK293T cells were transfected with SF-ZXDA or SF-Cyclin F and subjected to lysis and AP as in **(C)**. An empty vector (EV) was used as a negative control. Where indicated, the MLN4924 inhibitor was added to the cells 6 h prior to collecting. RRM2 is shown to interact with Cyclin F as a positive control. **(E)** FBXO38 localizes to the nucleus. HeLa cells were fixed, permeabilized, and incubated with an anti-FBXO38 antibody. DNA was stained with DAPI. Scale bar, 20 µm **(F)** ZXDA/B localizes to the nucleus. HeLa cells were treated as in **(F)** and incubated with an anti-ZXDA/B antibody. DNA was stained with DAPI. Scale bar, 20 µm. **(G)** Endogenous FBXO38 or endogenous ZXDA/B were immunoprecipitated from HEK293T cells using either FBXO38 or a ZXDA/B antibody. Non-specific IgG was used as a negative control. Whole-cell lysates (WCL) and immunoprecipitations (IP) were immunoblotted as indicated.

To identify substrates of SCF^FBXO38^, we performed affinity purification of the FBXO38 protein. Mass spectrometry analysis of the FBXO38 interactome revealed peptides corresponding to canonical subunits of SCF^FBXO38^ (SKP1, SUGT1, and CUL1) and previously identified FBXO38 interaction partner USP7 deubiquitinase (Zhang M. et al., 2008; Skaar et al., 2013; Georges et al., 2019). Additionally, the list of co-purified proteins contained a family of small DED-containing proteins DEDD and DEDD2, transcription co-regulator FAM172A, and zinc finger X-linked duplicated A and B proteins (ZXDA/B) (Figures 1A,B). ZXDA/B proteins were selected for further studies as their protein level increased upon FBXO38 knockdown, suggesting that they represent potential substrates of SCF^FBXO38^ ubiquitin ligase (Supplementary Figure S1A). ZXDA/B encoding genes evolved in two steps. First, *ZXDA* appeared as a retrogene from the *ZXDC* gene in placental mammals (Supplementary Figure S1B). Later, in the Boreoeutheria clade, *ZXDA* was duplicated, and *ZXDB* appeared in an inverted position on chromosome X (Supplementary Figure S1C). Translational products of *ZXDA* and *ZXDB* genes are almost identical, sharing 98% similarity in primary sequence (Supplementary Figure S1D; in the following text we will refer to endogenous proteins as ZXDA/B and exogenous recombinant protein as ZXDA). We screened a small panel of Cullin-RING ligase substrate receptors to confirm that the interaction between FBXO38 and ZXDA/B is specific (Figure 1C). In a complementary approach, we purified StrepII-FLAG-tagged ZXDA (SF-ZXDA) and found that endogenous FBXO38, CUL1, and SKP1, but not other tested F-box proteins, interacted specifically with ZXDA (Figure 1D). Finally, we confirmed that endogenous FBXO38 and ZXDA/B proteins localized into the nucleoplasm (Figures 1E,F, Supplementary Figure S1E) and mutually interacted (Figure 1G).

### FBXO38 Mediates Ubiquitination and Controls the Stability of ZXDA/B

**FIGURE 2:**
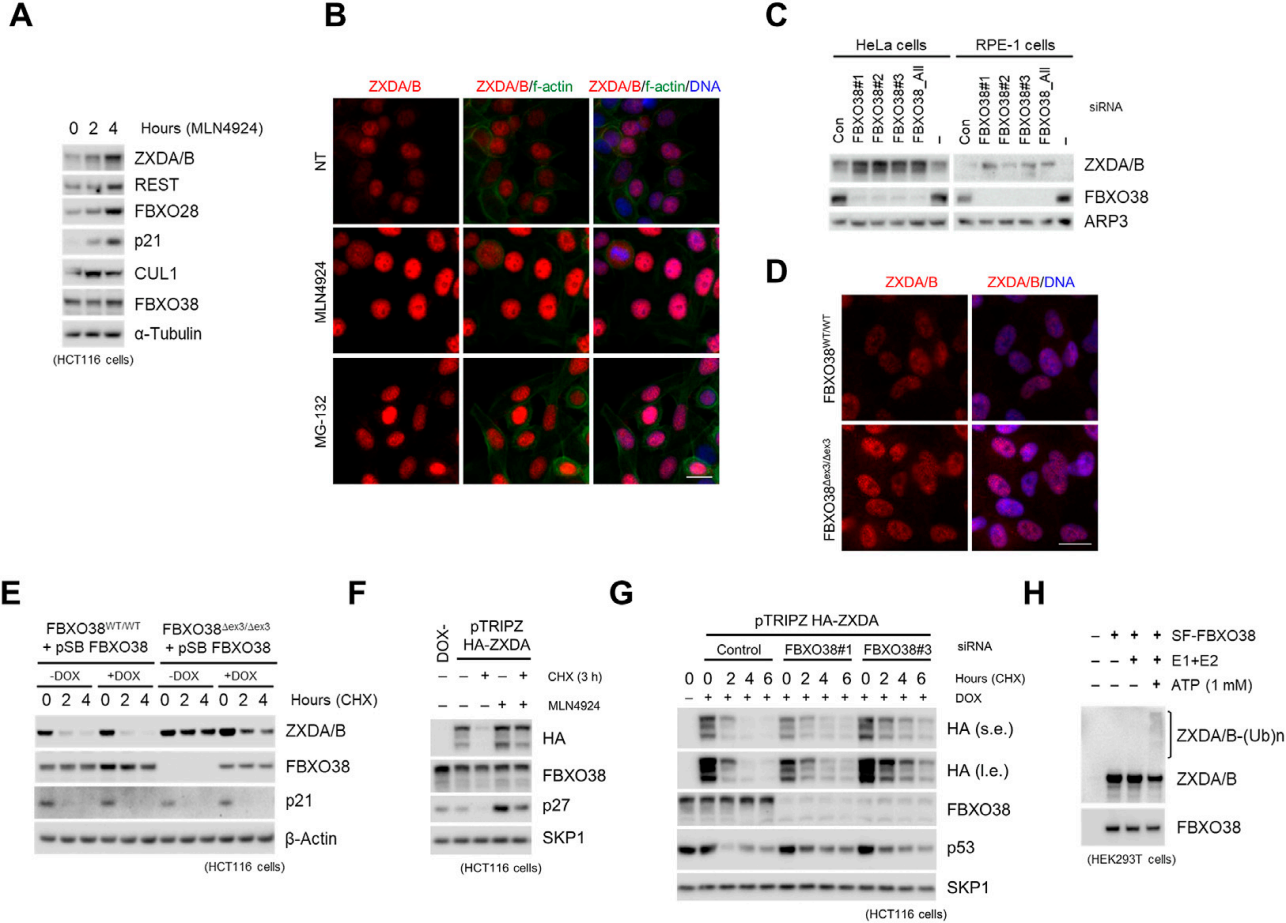
FBXO38 mediates ubiquitination and controls the stability of ZXDA/B. **(A)** HCT116 cells were treated with MLN4924 for 2 and 4 h, lysed and immunoblotted as indicated **(B)** HeLa cells were grown on slides and treated with MLN4924 or MG-132 for 12 h where indicated. The cells were fixed and immunostained with an anti-ZXDA/B antibody. DNA was stained with DAPI, and f-actin was visualized using phalloidin. Scale bar, 20 µm. **(C)** HeLa and RPE-1 cells were transfected with three different siRNAs targeting FBXO38, and whole-cell lysates were immunoblotted as indicated. Non-targeting siRNA (Con) or no siRNA were used as negative controls. **(D)** Wild-type (WT) or FBXO38 knockout RPE-1 cells (FBXO38^Δex3/Δex3^; KO) were grown on slides, fixed, and immunostained with the ZXDA/B antibody. DNA was stained with DAPI. Scale bar, 20 µm. **(E)** StrepII-FLAG-tagged FBXO38 (SF-FBXO38) was inducibly expressed in FBXO38 WT and KO HCT116 cells using the Sleeping Beauty Transposon System. Where indicated, the expression of FBXO38 was induced with doxycycline for 24 h. Cells were treated with cycloheximide (CHX) for the indicated time. Whole-cell lysates were immunoblotted as indicated. **(F)** HCT116 cells with doxycycline-inducible expression of HA-tagged ZXDA were treated with doxycycline for 24 h and then treated with CHX for 3 h. Where indicated, cells were pre-treated with MLN4924 for 3 h. Non-treated cells (DOX-) were used as a negative control. Whole-cell lysates were immunoblotted as indicated. **(G)** HCT116 cells with doxycycline-inducible expression of HA-tagged ZXDA were transfected with two different siRNAs targeting FBXO38. Expression of ZXDA was induced by doxycycline 18 h after transfection. Cells were treated with CHX for the indicated time and collected 48 h after transfection. Whole-cell lysates were immunoblotted as indicated **(H)** SF-FBXO38 was overexpressed in HEK293T cells and isolated using Strep-Tactin resin. Eluate was subjected to *in vitro* ubiquitination in the presence of E1 and E2 enzymes or E1, E2, and ATP. An empty vector (EV) was used as a negative control. ZXDA/B antibody was used to detect polyubiquitinated forms of co-purified endogenous ZXDA/B proteins.

To assess whether ZXDA/B proteins are targeted for protein degradation by Cullin-dependent ubiquitin ligases, we first utilized the neddylation inhibitor MLN4924, since NEDD8-cojugation to Cullin proteins is prerequisite for SCF activity (Baek et al., 2020). At two different time points after treatment, an increase in ZXDA/B protein levels was observed, similar to other well-characterized CUL1-dependent substrates REST, p21, and FBXO28 (Figure 2A) (Yu et al., 1998; Guardavaccaro et al., 2008; Cai et al., 2020). Upon neddylation or proteasome (MG-132) inhibition, the ZXDA/B proteins accumulated in the nuclear compartment specifically (Figure 2B). To test whether these effects are FBXO38-dependent, we silenced endogenous FBXO38 in HeLa and RPE-1 cell lines. Acute knockdown of FBXO38 led to the increased protein level of ZXDA/B compared to the control cells (Figure 2C). To further validate the effect of FBXO38 on ZXDA/B proteins stability, FBXO38^∆Ex3/∆Ex3^ HCT116 and RPE-1 cell lines lacking full-length protein were generated (FBXO38 knockout; KO). As expected, we observed an increased protein level of ZXDA/B in the nuclei of the FBXO38 KO cells (Figure 2D). By treating KO cells with translation inhibitor cycloheximide (CHX), we confirmed the increased stability of ZXDA/B in the absence of FBXO38. This effect was rescued by the expression of exogenous FBXO38, which led to the destabilization of ZXDA/B. At the same time, another target of CUL1-dependent degradation, p21, did not show any change in stability upon FBXO38 expression (Figure 2E). Using the HCT116 cell line with an inducible expression of exogenous ZXDA, we confirmed that these effects are transcriptionally independent. Both the neddylation inhibition and FBXO38 knockdown increased exogenous ZXDA protein level and stability (Figures 2F,G). Finally, affinity-purified FBXO38 promoted *in vitro* ubiquitination of endogenous ZXDA/B (Figure 2H). Overall, these results show that SCF^FBXO38^ controls ZXDA/B proteins stability via Cullin-dependent protein degradation.

### FBXO38 Associates With ZXDA/B via Zinc Finger Linker Motif

**FIGURE 3:**
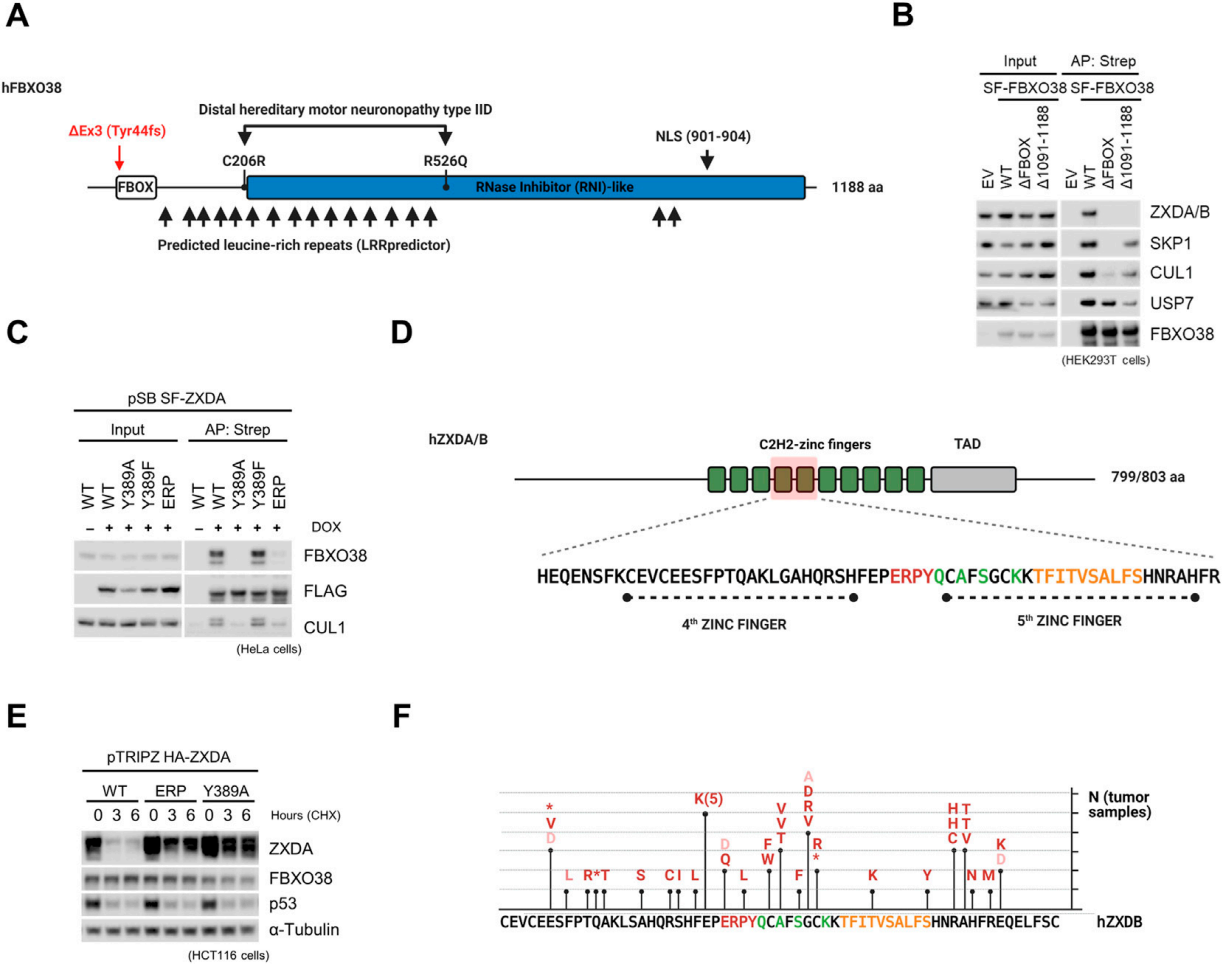
FBXO38 associates with ZXDA/B via the zinc finger linker motif. **(A)** A scheme illustrating human FBXO38 structural features and disease-associated mutations. The F-box motif is located in the N-terminus followed by predicted leucine-rich repeats. Locations of the two mutations (C206R and R526Q) found in patients with distal hereditary motor neuronopathy type IID are depicted. The previously confirmed nuclear localization sequence (NLS) is located in the C-terminal part. Red arrowhead indicates the site of frameshift in FBXO38^Δex3/Δex3^. **(B)** HEK293T cells were transfected with StrepII-FLAG-tagged wild-type FBXO38 (SF-FBXO38 WT), FBXO38 lacking the F-box motif (FBXO38^ΔFBOX^) or C-terminus (FBXO38^Δ1091-1188^), treated with MLN4924 6 h prior to collecting, lysed, and subjected to affinity purification (AP) using Strep-Tactin resin. Inputs represent 1% of the whole-cell lysates subjected to AP. **(C)** HeLa cells with the inducible expression of SF-ZXDA and its mutants were treated with doxycycline for 24 h where indicated. Whole-cell lysates were subjected to affinity purification (AP) using Strep-Tactin resin and immunoblotted as indicated. Inputs represent 1% of the whole-cell lysate subjected to AP. **(D)** A schematic representation of human ZXDA and ZXDB proteins. Both contain ten C2H2 Zinc fingers (green boxes) followed by a previously reported transcriptional activation domain (TAD) (Al-Kandari et al., 2007). The zinc fingers important for FBXO38-interaction are red-tinted. Amino acids encompassing this region are depicted below, with a zinc finger linker necessary for FBXO38-interaction highlighted in red. Mutations of amino acids marked in yellow had a negative effect on FBXO38 interaction (degron). The green-labeled amino acids differ between ZXDA/B and ZXDC. **(E)** HCT116 cells with inducible expression of HA-tagged ZXDA and its mutated forms were incubated in the presence of doxycycline for 24 h and then treated with cycloheximide (CHX) for the indicated time. **(F)** ZXDB mutations identified in tumor samples (The Cancer Genome Atlas cohort). Mutations found in the vicinity of the FBXO38-dependent degron are depicted (deleterious mutations—red; synonymous or conservative mutations—orange). The ZXDB sequence is outlined below with amino acids highlighted as in **(D)**.

FBXO38 contains three structurally different domains (Figure 3A and Supplementary Figure S3A). Although initially described as an F-box lacking the typical WD40 or leucine repeats (thus designated FBXO) (Jin et al., 2004), detailed analysis using the Leucine-rich-repeats (LRR) and AlphaFold predictors showed that it is rather a typical F-box protein containing leucine-rich repeats (FBXL) (Martin et al., 2020; Jumper et al., 2021). The F-box motif, essential for SCF complex formation, and C-terminal domain were indispensable for FBXO38 interaction with ZXDA/B. In contrast, the loss of these motifs did not affect the interaction with deubiquitinase USP7 (Figure 3B). Mutants of FBXO38 found in patients with muscular atrophy interacted with ZXDA/B more strongly, especially after MLN4924 treatment (Supplementary Figure S3B).

A series of ZXDA mutants immunopurifications revealed that FBXO38 recognizes a motif encompassing amino acid residues 386–408 of ZXDA (Supplementary Figure S3C–S3J). We chose Y389A and ERP386-388AAA mutants for further experiments (Figure 3C,D, Supplementary Figure S3K,L). Tandem purification of the FBXO38-ZXDA complex revealed that their interaction is independent of posttranslational modification of this motif, as we did not detect any modification in the identified peptides covering the essential region (Supplementary Figure S3M,N). Furthermore, we confirmed increased stability of selected mutants compared to wild-type ZXDA (Figure 3E). An *in silico* search revealed that only ZXDA/B/C proteins contain this motif (Supplementary Figure S3O,P). Importantly, we observed that ZXDC, heterodimer partner of ZXDA/B (Al-Kandari et al., 2007), interacted with FBXO38 only upon proteasome or neddylation inhibition and that this interaction correlated with ZXDA/B stabilization (Supplementary Figure S3Q,R). Moreover, the ZXDC protein level was not elevated upon the FBXO38 siRNA-mediated knockdown, and *in silico* homology modeling of ZXDA/B/C zinc fingers revealed that minor differences in the FBXO38 interacting region can affect the orientation of amino acids essential for the interaction (Supplementary Figure S3S,T) (Wang et al., 2018; Waterhouse et al., 2018).

Finally, an analysis of The Cancer Genome Atlas (TCGA) revealed that *ZXDA/B* are significantly mutated in several tumor types and that a large portion of these ZXDA/B cancer-associated mutations is located in the vicinity of the FBXO38-dependent degron (Figure 3F and Supplementary Figure S3U).

### ZXDA/B Do Not Act as Transcription Factors

**FIGURE 4:**
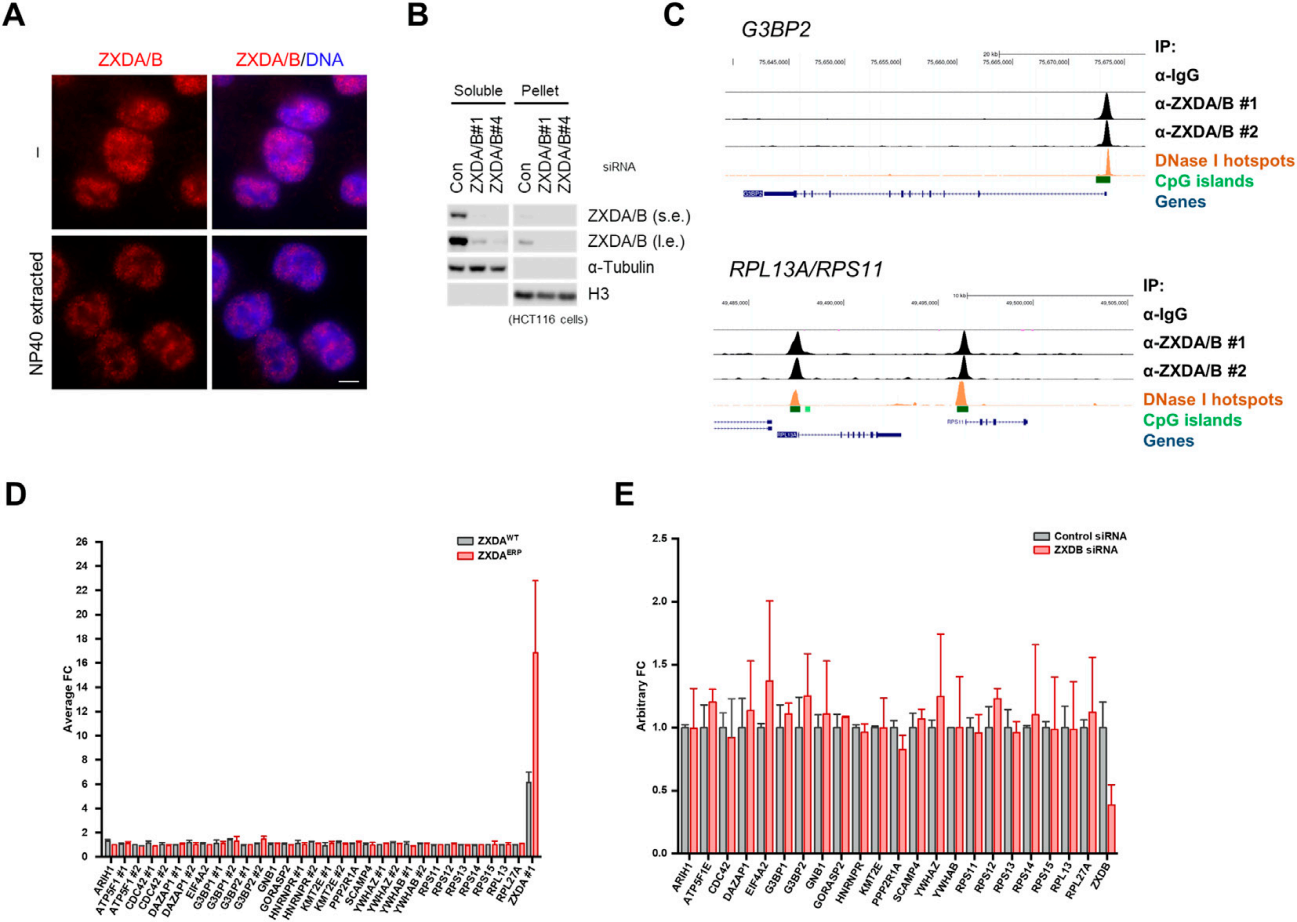
ZXDA/B do not act as transcription factors. **(A)** Representative images of DNA-associated ZXDA/B in HCT116 cells. Cells were either directly fixed or the soluble proteins were pre-extracted using a CSK buffer with 0.5% NP-40 for 10 min. DNA was visualized using DAPI. Scale bar, 5 µm **(B)** The HCT116 cells were subjected to isotonic lysis, soluble fractions were collected and the pellets were further lysed in the presence of Benzonase nuclease and subsequently heat denatured in the presence of 1% SDS for insoluble fractions. Comparable amounts of soluble and insoluble fractions were loaded and immunoblotted as indicated. Both sides of the panel represent the same exposure time; l. e. stands for long exposure, s. e. short exposure. **(C)** ChIP-seq tag profiles covering *G3BP2* (upper panel), *RPS11*, and *RPL13A* (lower panel) loci showing a high correlation between two independent ZXDA/B ChIP-seq samples. The upper tag profiles represent the control and two anti-ZXDA/B ChIP-seq samples. The lower panels show the location of the genes, DNase I hypersensitivity regions, and CpG islands. The scheme was generated using a UCSC Genome Browser (Kent et al., 2002). **(D)** Microarray analysis of gene expression in the HCT116 cell line with inducible expression of wild-type (WT) or mutant (ERP) ZXDA. The cells were incubated in doxycycline for 24 h. Only genes with ZXDB-associated promoters are shown. All numbers were compared to the mean value obtained in untreated (non-induced) control. The final value represents the mean calculated from two independent experiments. **(E)** RNA-seq analysis of the HCT116 cell line transfected with two different control siRNAs and two siRNAs targeting ZXDB. All counts (FKPM) were compared to the average count obtained in control samples. Only genes with ZXDB-associated promoters are shown.

To further understand ZXDA/B protein function, we determined whether its DNA-binding activity affects the transcriptional level of genes located nearby its binding sites. As already mentioned, ZXDA/B are nuclear proteins. Detergent pre-extraction or soluble fraction analysis via western blot showed that a significant portion of the ZXDA/B proteins is nucleoplasmic, not associated with chromatin (Figures 4A,B). To determine the DNA-binding specificity of ZXDA/B, we performed chromatin immunoprecipitation using an anti-ZXDA/B antibody followed by next-generation sequencing (ChIP-seq) in the HCT116 cells. An analysis of the normalized data showed that the ZXDA/B proteins bind the chromatin in numerous gene promoters. Most of the ZXDA/B-associated loci were located nearby transcription starting sites (Supplementary Table S1), and previously designated as CpG islands and DNase I hypersensitive sites (Figure 4C and Supplementary Figure S4A). Furthermore, the analysis of MACS2-called peaks using regulatory sequence analysis tools (RSAT) software revealed significant GC-enrichment in close vicinity to the peak summits (Supplementary Figure S4B,C) (Zhang Y. et al., 2008; Thomas-Chollier et al., 2012). ChIP-seq data and ZXDA/B binding specificity were confirmed using publicly available data from the Encode and GEO databases (GSE91872). Genes with strong enrichment of ZXDA/B in their promoters belong to several gene ontology groups, including genes encoding ribosomal and stress granule proteins (Supplementary Figure S4D). Surprisingly, neither overexpression of ZXDA (WT and ERP mutant) nor ZXDA/B knockdown showed any significant changes in the expression of genes with ZXDA/B-associated promoters (Figures 4D,E).

### ZXDA/B Are CENP-B Associated Proteins

**FIGURE 5:**
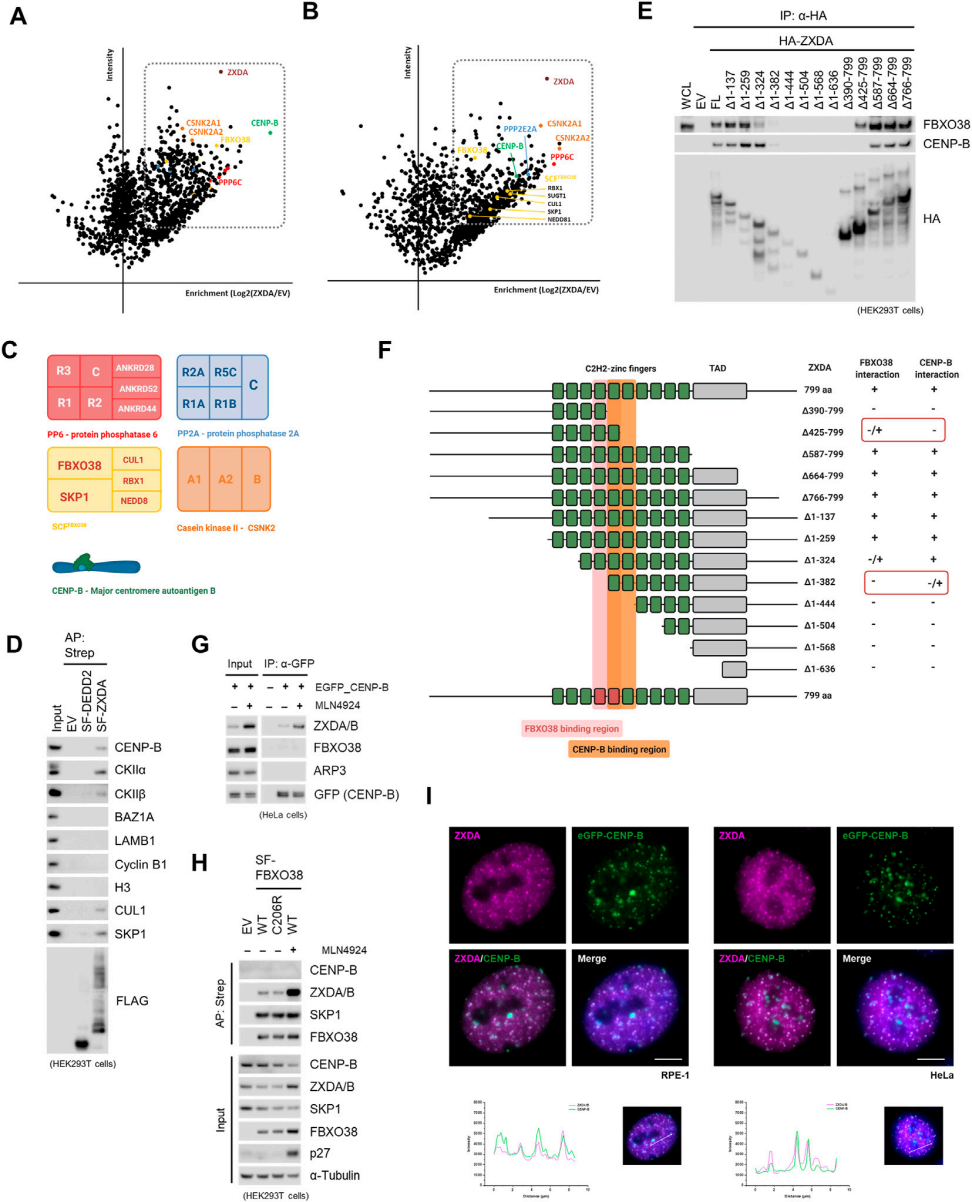
ZXDA/B are CENP-B associated proteins. **(A)** Schematic representation of mass-spectrometry identification of ZXDA-interacting proteins. Affinity-purified proteins from HEK293T cells expressing StrepII-FLAG-tagged ZXDA (SF-ZXDA) were identified by Liquid Chromatography with Tandem Mass-Spectrometry (LC-MS/MS). Proteins that were significantly enriched in ZXDA purifications compared to the controls are highlighted in box. The graph represents maximum intensity and the enrichment calculated from ratio of intensities of identified proteins. Colors represent the identity of proteins to different complexes as it is depicted in **(C)**. **(B)** Schematic representation of mass-spectrometry identification of ZXDA-interacting proteins. Affinity purified proteins from HeLa cells with inducible expression of SF-ZXDA were identified by LC-MS/MS as in **(A)**. **(C)** Schematic composition of proteins and protein complexes enriched in ZXDA interactome from **(A)** and **(B)**. **(D)** HEK293T cells were transfected with SF-tagged ZXDA or DEDD2, and whole-cell lysates were subjected to affinity purification (AP) and subsequently immunoblotted as indicated. An empty vector (EV) was used as a negative control. The input represents 1% of the lysate subjected to AP. **(E)** HEK293T cells were transfected with HA-tagged full-length (FL) ZXDA or its deletion variants. Cells were treated with the MLN4924 inhibitor 6 h prior to collecting. Whole-cell lysates (WCL) were subjected to immunoprecipitation (IP) using anti-HA beads. Empty vector (EV) was used as a negative control. **(F)** Schematic representation of full-length ZXDA (1–799 aa) or its N- and C-terminally truncated variants. Zinc fingers are depicted as green boxes, those essential for interaction with FBXO38 are red-tinted, with CENP-B are orange-tinted. The middle panel indicates deleted amino acid positions in ZXDA. Interacting variants are indicated by the symbol (+) (+/-) denotes reduced binding, and (-) denotes a lack of binding. TAD; transcriptional activation domain. **(G)** HeLa cells expressing eGFP-CENP-B were treated with MLN4924 for 6 h where indicated. Soluble protein fraction was subjected to immunoprecipitation (IP) using GFP-Trap magnetic beads. Parental cells were used as negative control. Input represents 1% of the lysate subjected to IP. **(H)** HEK293T cells were transfected with WT or mutant (C206R) SF-FBXO38 and subjected to lysis and AP. An empty vector (EV) was used as a negative control. Where indicated, the MLN4924 inhibitor was added to the cells 16 h prior to collecting. p27 expression is shown as a positive control for neddylation inhibition. **(I)** Representative images of RPE-1 (left panel) and HeLa (right panel) cells with inducible expression of SF-ZXDA and stable expression of eGFP-CENP-B. The cells were incubated in the presence of doxycycline for 3 h. Scale bar, 5 µm.

To clarify the biological function of ZXDA/B proteins, a series of affinity purifications of ZXDA were performed to determine its interactome in stably transfected HeLa cells and transiently transfected HEK293T cells. First, whole-cell lysates were prepared with turbonuclease to include chromatin-associated proteins. Subsequently, proteins co-purified with ZXDA were identified by mass-spectrometry analysis. In addition to the SCF^FBXO38^ subunits, ZXDA was associated with casein kinase 2 (CK2), protein phosphatase 2/6, and centromere protein B (CENP-B) (Figures 5A–C). By small-scale immunopurification, we confirmed that CENP-B, as well as CK2α and CK2β, interacted specifically with ZXDA and not with another interactor of FBXO38, DEDD2, that was used as a negative control (Figure 5D). As CENP-B is rarely found as a contaminant in mass-spectrometry analyzed purifications, we aspired to verify its interaction with ZXDA/B further. We revealed that the fifth and sixth zinc fingers of ZXDA protein are required for the interaction with CENP-B (Figure 5E). A comparison of the interaction profile of FBXO38 and CENP-B with ZXDA revealed that they bind overlapping regions (Figure 5F). Next, we established HeLa cells stably expressing eGFP-tagged CENP-B. We revealed that endogenous ZXDA/B co-precipitated with GFP-tagged CENP-B and that their interaction is enhanced by neddylation inhibition, most probably because ZXDA/B proteins level increased upon neddylation inhibition. Interestingly, FBXO38 did not interact with CENP-B, suggesting that their binding to ZXDA/B is mutually exclusive (Figure 5G). This observation was further confirmed by analysis of proteins co-purified with FBXO38 (Figure 5H).

Finally, to answer the question whether ZXDA/B are constitutive centromeric proteins, we analyzed different cancer cell lines using immunofluorescence. We did not observe any enrichment of endogenous ZXDA/B in distinct centromeric spots (data not shown). This result was not surprising, as we did not detect any centromeric chromatin DNA enrichment in ZXDA/B ChIP-seq. However, we found that brief inducible expression of exogenous ZXDA transiently colocalized in centromeric regions marked by eGFP-tagged CENP-B (Figure 5I).

### FBXO38 Controls Centromeric Signature via ZXDA/B Stability

**FIGURE 6:**
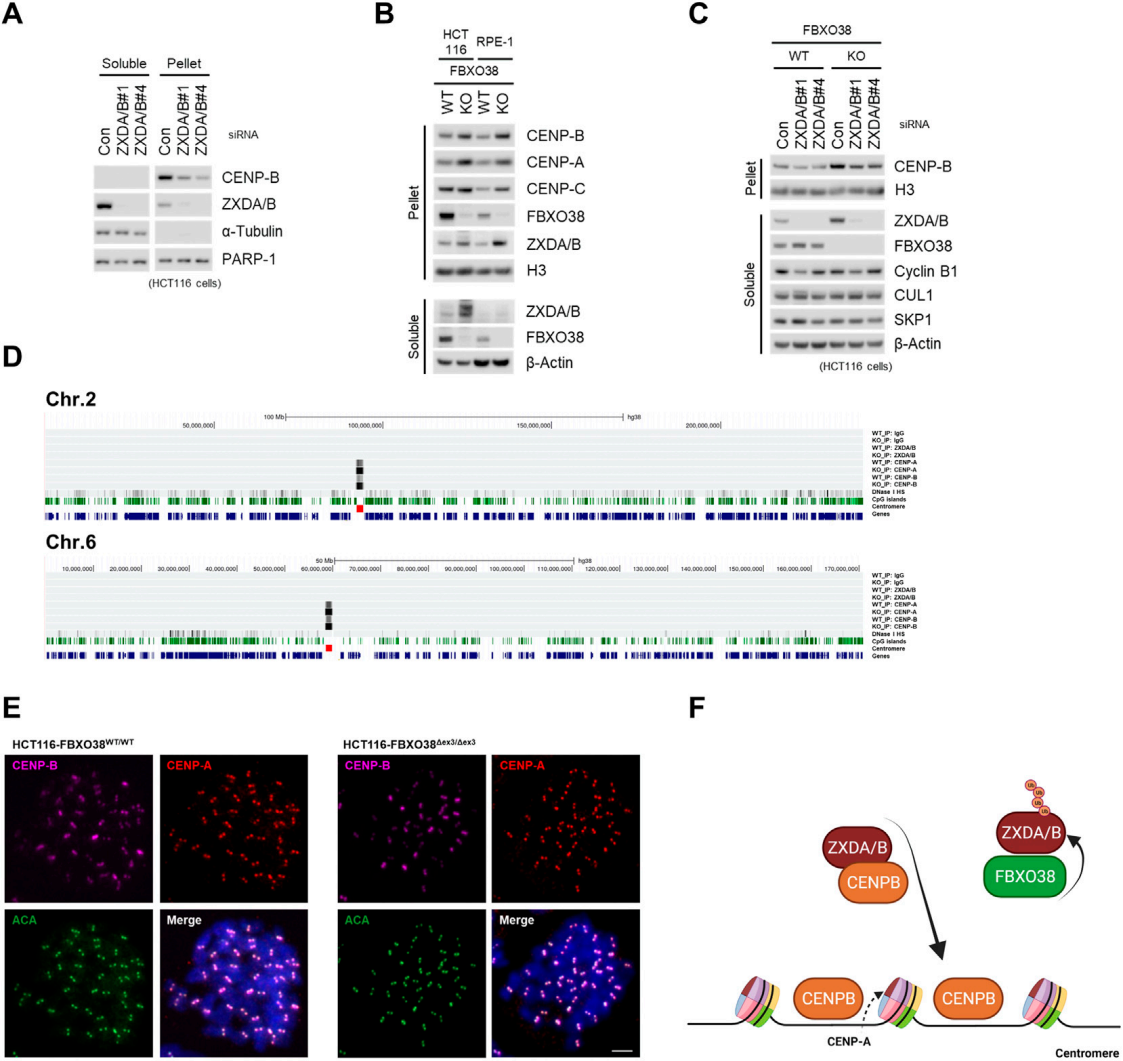
FBXO38 controls centromeric signature via ZXDA/B stability. **(A)** HCT116 cells were transfected with two different siRNAs targeting ZXDA/B, or with control siRNA. The cells were subjected to isotonic lysis 48 h after transfection, soluble fractions were collected and the pellets were further lysed in the presence of Benzonase nuclease and subsequently heat denatured in the presence of 1% SDS for insoluble fractions. Lysates were immunoblotted as indicated **(B)** FBXO38 wild type (WT) and knockout (KO) HCT116 or RPE-1 cells were subjected to fractionation as in **(A)** and immunoblotted as indicated. **(C)** FBXO38 WT and KO HCT116 cells were transfected with two different siRNAs targeting ZXDA/B, or with control siRNA, lysed and immunoblotted as in **(A)**. The longer exposure of CENP-B immunostaining is available as Supplementary Figure S6A. **(D)** ChIP-Seq analysis of chromatin-bound proteins in FBXO38 WT and KO HCT116 cell line. Fragmented chromatin was immunopurified with either non-specific IgG, or ZXDA/B, CENP-A, and CENP-B polyclonal antibodies. Trimmed reads were aligned using the Bowtie2 program and further normalized with bamCoverage utility (Galaxy—DeepTools) to obtain counts per million (CPM) values. Coverage of the human genome (hg38) was visualized in the UCSC genome browser. The figure shows two chromosomes (2 and 6) coverage with other chromosomes depicted in (Supplementary Figure S6D). The quantity of precipitated chromatin is shown as a density graph using autoscale to highlight the centromeric enrichment. The single lines below the chromosome range ruler represent ChIP results from IgG control, ZXDA/B, CENP-A, and CENP-B (in FBXO38 WT and KO HCT116 cells). These lines are followed by a density graph of DNase I hypersensitivity, CpG islands (green), centromere (red), and genes locations. **(E)** Representative images of a mitotic spread of FBXO38 WT (left) and KO (right) HCT116 cell line. The cells were pre-treated with colchicine and spun onto slides. Permeabilized cells were fixed and immunostained with anti-centromere antibodies (ACA) and anti-CENP-A and CENP-B antibodies. Chromosomes were visualized with DAPI. Scale bar, 5 µm **(F)** Model of FBXO38-dependent regulation of the ZXDA/B protein. ZXDA/B proteins controls centromeric CENP-B protein level. FBXO38 is responsible for ZXDA/B degradation and limits CENP-B chromatin loading.

To clarify the physiologic role of ZXDA/B in centromeric chromatin control, we assessed the impact of their knockdown on the CENP-B protein level. Independent siRNAs targeting ZXDA/B significantly decreased the CENP-B protein level in chromatin (pellet) fraction (Figure 6A). Next, we assessed the effect of FBXO38 depletion on CENP-B expression and localization. As expected, FBXO38 deficiency led to increased levels of the ZXDA/B protein in immortalized RPE-1 and HCT116 cells. Accordingly to our previous observation, ZXDA/B acted as positive regulators of centromeric chromatin, and FBXO38 deficiency increased CENP-A, -B, and -C protein levels (Figure 6B). Our next goal was to rescue the effect of FBXO38 knockdown with simultaneous ZXDA/B inactivation. Since constitutive ZXDA/B KO cells were not viable, we proceeded with an acute knockdown of their expression using siRNAs. This inactivation led to the rescue of the CENP-B protein to the control level in the FBXO38-deficient HCT116 cells. To exclude cell cycle-related artifacts, the expression of cyclin B was assessed. Despite a slight change in the level of cyclin B in cells transfected with one of the ZXDA/B siRNAs, the pattern did not generally correlate with any significant cell cycle disruption (Figure 6C and Supplementary Figure S6A). Subsequently, we focused on the subgenomic localization of centromeric chromatin proteins. As it was shown that both CENP-A and CENP-B proteins could be deposited into extra-centromeric chromatin, such as neocentromeres, DNA damage sites, and transcription factor hotspots (Zeitlin et al., 2009; Scott and Sullivan, 2014; Athwal et al., 2015), we were interested in whether the loss of FBXO38 leads to these aberrations. For this purpose, ChIP-seq analyses of endogenous CENP-A/B proteins were performed. In FBXO38-deficient HCT116 cells, we did not detect any enrichment of previously described ZXDA/B binding sites in ChIP using CENP-A/B antibodies (Supplementary Table S1; examples in Supplementary Figure S6B), nor did we observe any CENP-A/B binding to the non-centromeric regions described previously (examples in Supplementary Figure S6C) (Athwal et al., 2015). Moreover, ChIP-seq results showed that the FBXO38 deficiency in HCT116 cells led to significant upregulation of CENP-A/B proteins in centromeric regions across the genome (Figure 6D, Supplementary Figure S6D). Finally, we visualized the subchromosomal localization of centromeres by immunofluorescence staining of CENP-A/B proteins in mitotic cell spreads. Both FBXO38 WT and KO cells had the same number of chromosomes (∼45) and we observed no ectopic centromere formation (Figure 6E). These results suggest that the endogenous ZXDA/B proteins act as positive regulators of chromatin CENP-B protein loading and thus represent previously unknown controllers of the epigenetic signature of the centromeres. Finally, we proved that FBXO38-dependent ZXDA/B degradation regulates this process and controls the composition of centromeric chromatin and integrity (Figure 6F).

## Discussion

Here we show that FBXO38 ubiquitin ligase substrate receptor interacts with the zinc finger proteins ZXDA and ZXDB and targets them for degradation. FBXO38 recognizes a zinc finger linker in ZXDA/B, which represents a consensus motif found in many commonly expressed zinc finger proteins. Surprisingly, none of them had ever been detected in any of our proteomic analyses of FBXO38-associated proteins, indicating the hidden complexity of how FBXO38 recognizes this degron. Interestingly, we found that ZXDC, a very close homolog of ZXDA/B that shares an identical motif in the zinc finger linker, did not interact with FBXO38. Homology modeling revealed a significant difference in the structural positioning of essential tyrosine due to different amino acid compositions surrounding this motif. Although these *in silico* results support our *in vitro* observations, we cannot exclude the possibility that FBXO38 also controls ZXDC under certain conditions. Furthermore, we proved that phosphorylation of this motif is not required, but it is also plausible that phosphorylation of degron residues and surrounding amino acids may have an inhibitory impact on the interaction between ZXDA/B and FBXO38. Similarly positioned tyrosine in the Yin Yang 1 protein (YY1) is the target of Src tyrosine kinases, and its phosphorylation interferes with the DNA and RNA binding of YY1 protein (Wang and Goff, 2015). Importantly, threonine and serine residues adjacent to C2H2 zinc finger linkers are globally phosphorylated during mitosis (Rizkallah et al., 2011). This phosphorylation was shown to be inhibitory towards their DNA-binding and represents cellular control of the transcription factor function during mitosis (Dovat et al., 2002). Interestingly, ZXDA/B proteins contain glutamate residue at the corresponding position (hZXDA/B Glu384/388), indicating that these zinc fingers may not be involved in DNA binding since glutamate mimics phosphorylated threonine.

The ZXDA/B/C proteins were shown to act as transcriptional regulators (Al-Kandari et al., 2007). Here we demonstrated that ZXDA/B proteins are in complex with CENP-B, an enigmatic DNA-binding centromeric protein controlling the centromere integrity and preventing aneuploidy (Fachinetti et al., 2015; Gamba and Fachinetti, 2020). We propose that the interaction of ZXDA/B with FBXO38 and CENP-B is mutually exclusive since we have neither detected the CENP-B protein in FBXO38 purifications nor observed FBXO38 in CENP-B pulldowns. The underlying reason could be the partial overlap of FBXO38 and CENP-B interaction motifs in ZXDA/B. Although we have not studied the region in CENP-B required for ZXDA/B binding, we observed that they interact preferentially in the soluble fraction. This observation is supported by the fact that we have not seen localization of endogenous ZXDA/B in centromeric regions, nor have we observed precipitation of centromeric chromatin using the ZXDA/B antibody. In fact, ZXDA colocalizes with CENP B only transiently after a brief induction of exogenous ZXDA expression.

We proposed that ZXDA/B proteins positively affect CENP-B loading into the centromere region. ZXDA/B stabilization in FBXO38 KO cells led to increased CENP-A/B/C protein levels. There is growing evidence that centromere chromatin is dynamically regulated upon stress conditions, viral infection, or during the differentiation process (Everett et al., 1999; Hedouin et al., 2017; Hou et al., 2021). However, we do not currently understand the cellular mechanisms governing FBXO38/ZXDA/B-dependent regulation of centromeric chromatin. In FBXO38-deficient cell lines, FBXO38 appears to control steady-state levels of ZXDA/B proteins, but we cannot rule out the possibility of a signaling pathway controlling this interaction. In addition, the physiological role of human FBXO38 seems unclear. FBXO38-deficient mice are growth-retarded with a defect in the maturation of Sertoli cells, the postmitotic somatic population required for germ cell nurturing. FBXO38 deficiency leads to ZXDA/B stabilization and increased centromeric CENP-A/B protein levels in Sertoli cells. As a result, mouse testes exhibit disorganized spermatogenesis and decreased fertility (Dibus et al., 2022). Another hint for the physiological role of FBXO38 comes from discoveries that several mutations in the FBXO38 gene drive early-onset distal muscular dystrophy (Sumner et al., 2013; Akcimen et al., 2019). Increased anterior horn motor neuron loss was observed in the spinal cord of these patients. Although it was not our aim to identify the role of FBXO38 in neural tissue, ZXDA/B proteins and centromeres can be deregulated in these patient motor neurons similarly to cancer cell lines or the previously mentioned Sertoli cells. This biochemical pathway could thus link centromere integrity to the development of a subset of neurodegenerative syndromes.

Since ZXDA/B/C nuclear factors have previously been identified as transcriptional regulators, we expected that their binding to DNA would affect the expression of nearby genes (Al-Kandari et al., 2007; Aleksandrova et al., 2010). However, we did not observe any differences in the expression of these genes after ZXDA-inducible overexpression or ZXDA/B siRNA-mediated knockdown. This could be due to a functional uncoupling of the ZXDA/B proteins from the transcription machinery or because the effect of ZXDA/B expression on transcription is not immediate. Unfortunately, we were unable to generate CRISPR-mediated ZXDA/B knockdown (in mouse or cancer cell lines) and thus analyze the long-term effects of ZXDA/B expression. Finally, ZXDA/B might have context-specific partners that would affect the transcription of downstream genes, but we are currently unaware of any such ZXDA/B interaction partners.

The frequency of CENP-A positive nucleosomes in centromere chromatin is tightly regulated and dependent on various factors, including CENP-B itself, as shown in human artificial chromosome experiments (Ohzeki et al., 2002). Human epithelial diploid cells have been reported to contain ∼400 molecules of CENP-A in their centromeres. This observation is in striking contrast to a large number of CENP-B box-containing satellite repeats in the human genome, particularly in centromeric regions (Bodor et al., 2014; McNulty and Sullivan, 2018). Such paradox suggests that the CENP-B box is essential but not sufficient for CENP-A nucleosome *de novo* assembly, and there have to exist controlling mechanisms involved in this process. Our results suggest that the FBXO38-ZXDA/B biochemical pathway could be one of these mechanisms, as we demonstrated that FBXO38 KO cells exhibited stabilized centromeric chromatin proteins. To understand the nature of this stabilization, we performed a ChIP-seq analysis to clarify differences in CENP-A/B proteins DNA interaction upon the loss of FBXO38. As shown previously, CENP-A centromeric histone can be transiently present at the DNA damage sites and transcription factor hotspots (Zeitlin et al., 2009; Scott and Sullivan, 2014; Athwal et al., 2015). This might suggest that FBXO38 controls centromere chromatin deposition at these sites, potentially blocking the formation of neocentromeres. However, we did not observe any ectopic CENP-A/B proteins in FBXO38 knockout cells, implying that ZXDA/B controls the deposition of these chromatin proteins primarily in the centromeres themselves.

Interestingly, a similar complex, centromere binding factor 3 (CBF3), controls the centromere chromatin establishment and kinetochore assembly in budding yeast (Lechner and Carbon, 1991). The point centromere in budding yeast consists of three short DNA elements CDE I, CDE II and CDE III, and the CBF3 complex binds to the CCG motif in CDE III. CBF3 complex consists of leucine-rich repeats containing F-box proteins Ctf13, Skp1^SKP1^, zinc finger protein Cep3, and the kinetochore protein Ndc10 (Yan et al., 2018). In addition, Skp1 chaperone Sgt1^SUGT1^ is also essential for the function of the CBF3 complex (Kitagawa et al., 1999). Importantly, the CBF3 complex controls centromere chromatin loading of Cbf1^CENP−B^ and Cse4^CENP−A^, similarly to FBXO38/ZXDA/B complex (Hemmerich et al., 2000; Zhang et al., 2007). However, there are no identified mammalian homologs for Ctf13 ubiquitin ligase and Cep3 zinc finger protein, and the similarity to FBXO38/ZXDA/B is based on structural and functional protein motifs. Moreover, we did not observe a tertiary complex between CENP-B/ZXDA/B and FBXO38, and FBXO38 did not localize to nor interact with centromeric DNA.

Aberrant centromere chromatin composition frequently results in improper kinetochore formation and aneuploidy, one of the hallmarks of cancer (Vasudevan et al., 2021). As previously mentioned, CENP-B protein is required for faithful chromosome segregation (Fachinetti et al., 2015). On the other hand, increased CENP-B expression leads to its non-specific DNA binding (Tachiwana, Miya et al., 2013) and the consequent increase in chromatin CENP-A and CENP-C levels (Okada et al., 2007; Fachinetti et al., 2015). Importantly, overexpression of CENP-A has been shown to affect the fidelity of mitosis and promote tumorigenesis (Tomonaga et al., 2003; Jeffery et al., 2021). Given that such misexpression of centromeric chromatin may lead to chromosomal aberrations and possibly cancer-related malformations, there is a possibility that FBXO38 may provide a fine-tuning mechanism to avoid these anomalies. In support of this hypothesis, it has been shown that FBXO38 prevents cytokinesis and mitotic defects and that cancer cells lacking FBXO38 exhibited an increased frequency of multinucleated cells (Georges et al., 2019).

## Materials and Methods

### Cell Culture Procedures

Human cell lines HEK293T (female), hTERT RPE-1 (female), HCT 116 (male), U-2 OS (female), HeLa (female) were maintained in Dulbecco’s Modified Eagle’s Medium (DMEM) supplemented with 10% fetal bovine serum (FBS) and penicillin, streptomycin, and gentamicin. All cell lines were cultured in a humidified incubator at 37°C with 5% CO_2_. Cycloheximide (100 μg/ml), MLN4924 (2 µM), or MG-132 (10 µM) were used where indicated. If not stated otherwise, doxycycline hyclate (Sigma Aldrich) was used at 1 μg/ml concentration. Cells were regularly screened for *Mycoplasma* contamination.

### Plasmid Construction

cDNAs of human *FBX O 38* (Origene #RC204380), *ZXDA* (TransOMIC Technologies, #BC059356), or *ZXDC* (kind gift from Dr. Joseph D. Fontes.) genes were cloned into pcDNA3.1 containing either N-terminal twinStrepII-FLAG-tag (pcDNA3.1—NSF) or an N-terminal hemagglutinin-tag (pcDNA 3.1—HA). For mutated and truncated variants of these genes, PCR mutagenesis was carried out using primers designed with QuikChange^®^ Primer Design Program (Agilent) with subsequent DpnI digestion. All the constructs created were verified by sequencing. For inducible expression, cDNAs of selected genes were cloned into lentiviral pTRIPZ™ (Dharmacon) or pSBtet vectors. A retroviral construct expressing eGFP-CENPB was a gift from Iain Cheeseman (pKG141-eGFP-CENPB; Addgene plasmid #69759). The cDNA library of the ubiquitin ligases was prepared as described previously (Lidak et al., 2021).

For FBXO38^∆Ex3/∆Ex3^ cell line generation (HCT116 and RPE-1 FBXO38 KO), single guide RNAs (sgRNAs) were cloned into the LentiCRISPR (pXPR_001; Addgene, #49535) containing dual sgRNA and hSpCas9 expression cassettes. Optimal sgRNA sequences were designed using http://crispr.mit.edu/and the targeted sequences were: AAG​CTG​TAT​GAC​CGT​ATG​TGT​GG, GAT​GCA​TGT​TTT​CCG​GTG​AAT​GG. Successful deletion was confirmed by PCR and sequencing.

### Transient Transfections

HEK293T cells were transfected using polyethylenimine (PEI MW 25000, Polysciences). All other cell lines were transfected using Lipofectamine 2000 (Invitrogen) according to the manufacturer’s protocol. For transient transfections, the cells were collected 48 h after transfection. Where indicated, MLN4924 or MG-132 was used.

### Stable Cell Line Generation

For lentiviral particles production, HEK293T cells were co-transfected with the lentiviral vector containing HA-tagged ZXDA (pTRIPZ_HA-ZXDA) along with pCMV-dR8.2 (Addgene, #8455) and pCMV-VSV-G (Addgene, #8454). The lentiviral media was collected 48 h after transfection, mixed with Polybrene (Sigma Aldrich), and used for infecting the cells of interest. The stable clones were selected using puromycin.

For the transposition-based gene transfer, the cells were transfected with pSBtet containing SF-tagged ZXDA (pSB_NSF-ZXDA) along with the transposase-containing pSB100X. The selection of stable clones was carried out using puromycin 24 h after transfection.

HeLa cells stably expressing eGFP-CENP-B were generated by co-transfection of pKG141-eGFP-CENPB along with pCMV-VSV-G (Addgene, #8454) and gag/pol plasmid (Addgene, #14887) into HEK293T cells. The viral containing media was collected 48 h after transfection and used for infecting HeLa cells. CENP-B-expressing clones were selected according to eGFP positivity.

To generate FBXO38 knockout cell lines, the LentiCRISPR containing sgRNA targeting sequences were transfected into the previously subcloned HCT116 and RPE-1 cells. Two days after transfection, the cells were briefly selected with puromycin and sorted to get single-cell clones. Genomic DNA was extracted using a DNA-isolation buffer (0.2% Tween, 0.2% Triton-X100, Proteinase K 200 μg/ml) and genotyped by PCR using primers surrounding the targeted sequence. The PCR products were purified and sequenced to confirm the deletion.

### Gene Silencing

Small interfering RNAs (Sigma Aldrich) were transfected into the subconfluent cell lines using Lipofectamine RNAiMAX (Invitrogen) according to the manufacturer’s protocol. The sequences of the oligonucleotides targeting FBXO38 were GGG​UGU​AUU​UCA​GCG​AGU​AUU (#1), GGA​CUC​GAU​UGG​UUG​AUA​UUU (#2), and GAG​CGA​AGC​UGU​UUG​AGU​AUU (#3). The sequences of the oligonucleotides targeting ZXDA/B were GCU​CUG​UGG​UGU​UGG​AUA​AUU (#1), CUG​AAA​GGC​CAC​AGC​AUA​AUU (#2), CCA​AGA​AGC​ACC​AGC​UGA​AUU (#3), and ACA​CAU​AAG​UCU​UGG​AAA​UUU (#4). Non-targeting siRNAs were used as negative controls.

### Immunoblotting

For whole-cell lysates, cells were washed with ice-cold PBS and lysed in a lysis buffer (150 mM NaCl, 50 mM Tris pH 7.5, 0.4% Triton X-100, 2 mM CaCl2, 2mM MgCl2, 1 mM EDTA) in the presence of phosphatase a protease inhibitor cocktail (MedChem Express) and Benzonase nuclease (0.125 U/μl; Santa Cruz) for 30 min on ice. Lysates were mixed with an equal volume of 2% SDS in 50 mM Tris-HCl, pH 8, heated for 5 min in 95°C, and cleared by centrifugation. For soluble fraction, washed cells were lysed for 10 min in the same buffer without Benzonase nuclease, centrifuged for 5 min, 3,000 rpm, and the fraction was separated. The leftover insoluble fraction was further lysed for 15 min in the same buffer with the addition of Benzonase nuclease (1.25 U/μl), mixed with 2% SDS in 50 mM Tris-HCl, pH 8, to the final concentration 1%, heated for 5 min in 95°C and cleared by centrifugation.

Protein concentration was determined by the BCA method (Thermo Fisher Scientific). Samples were prepared by mixing with Bolt™ LDS Sample Buffer (Thermo Fisher Scientific) supplemented with 10% β-mercaptoethanol (Sigma Aldrich), heated for 5 min at 95°C, and then separated using NuPAGE™ 4–12% gradient Bis-Tris gel (Invitrogen). Afterward, the samples were transferred to PVDF membrane (Amersham), blocked with 5% milk (ChemCruz), and incubated with indicated antibodies diluted in 3% BSA (PanReac Applichem) in TBS-T overnight at 4°C. The HRP-conjugated secondary antibodies (Cell Signaling) were diluted in 5% milk in TBS-T. The membranes were developed using WesternBright ECL (Advansta) or SuperSignal™ West Femto Maximum Sensitivity Substrate (Thermo Fisher Scientific).

### Protein Immunoprecipitation and Affinity Purification

The cells were collected and lysed in the lysis buffer (150 mM NaCl, 50 mM Tris pH 7.5, 0.4% Triton X-100, 2 mM CaCl2, 2 mM MgCl2, 1 mM EDTA, supplemented with phosphatase and protease inhibitors) in the presence of Benzonase nuclease (0.125 U/μl; Santa Cruz) for 30 min on ice. The lysates cleared by centrifugation were then incubated with either Strep-Tactin^®^ Sepharose resin (IBA Lifesciences) for SF-tagged proteins or with Pierce™ anti-HA Magnetic Beads (Thermo Fisher Scientific) or GFP-Trap^®^ Magnetic Beads (ChromoTek) Purified Strep-tagged proteins were then eluted by desthiobiotin using Buffer E (IBA Lifesciences) and subsequently prepared for immunoblotting as described above. Elution of immunoprecipitated HA-tagged and eGFP-tagged proteins was carried out with 1x Bolt™ LDS Sample Buffer (Thermo Fisher Scientific). Eluates were then supplemented with β-mercaptoethanol (Sigma Aldrich) and incubated at 95 °C for 5 min. For immunoprecipitation of endogenous proteins, lysates were incubated with the indicated antibodies and mixed with Dynabeads^®^ Protein G. Rabbit IgG was used as a negative control. Elution of immunoprecipitated proteins was carried out under the same conditions as for anti-HA immunoprecipitation. For tandem affinity purification, HEK293T cells were co-transfected with constructs encoding SF-tagged and HA-tagged proteins. Cells were collected and lysed in the same lysis buffer as described above. The lysates were incubated with Strep-Tactin^®^ Sepharose resin and eluted as described above. A portion of the eluate was subsequently used for HA-immunoprecipitation. Precipitated proteins were eluted by 3M NaSCN.

### Mass Spectrometry

Protein Digestion. Desthiobiotine and NaSCN eluates were acetone precipitated and resuspended in 100 mM TEAB containing 1% SDC. Cysteins were reduced with 5 mM final concentration of TCEP (60°C for 60 min) and blocked with 10 mM final concentration of MMTS (10 min Room Temperature). Samples were cleaved on beads with 1 µg of trypsine at 37°C overnight. After digestion samples were centrifuged and supernatants were collected and acidified with TFA to 1% final concentration. SDC was removed by extraction to ethylacetate (Masuda et al., 2008). Peptides were desalted using in-house made stage tips packed with C18 disks (Empore) according to (Rappsilber et al., 2007).

nLC-MS 2 Analysis. Nano Reversed phase column (EASY-Spray column, 50 cm × 75 µm ID, PepMap C18, 2 µm particles, 100 Å pore size) was used for LC/MS analysis. Mobile phase buffer A was composed of water and 0.1% formic acid. Mobile phase B was composed of acetonitrile and 0.1% formic acid. Samples were loaded onto the trap column (Acclaim PepMap300, C18, 5 μm, 300 Å Wide Pore, 300 μm × 5 mm, 5 Cartridges) for 4 min at 15 μl/min. Loading buffer was composed of water, 2% acetonitrile and 0.1% trifluoroacetic acid. Peptides were eluted with Mobile phase B gradient from 4 to 35% B in 60 min. Eluting peptide cations were converted to gas-phase ions by electrospray ionization and analyzed on a Thermo Orbitrap Fusion (Q-OT-qIT, Thermo). Survey scans of peptide precursors from 400 to 1,600 m/z were performed at 120K resolution (at 200 m/z) with a 5 × 10^5^ ion count target. Tandem MS was performed by isolation at 1, 5 Th with the quadrupole, HCD fragmentation with normalized collision energy of 30, and rapid scan MS analysis in the ion trap. The MS 2 ion count target was set to 10^4^ and the max injection time was 35 ms. Only those precursors with charge state 2–6 were sampled for MS 2. The dynamic exclusion duration was set to 45 s with a 10 ppm tolerance around the selected precursor and its isotopes. Monoisotopic precursor selection was turned on. The instrument was run in top speed mode with 2 s cycles (Hebert et al., 2014).

Data analysis. All data were analyzed ans quantified with the MaxQuant software (version 1.5.3.8) (Cox et al., 2014). The false discovery rate (FDR) was set to 1% for both proteins and peptides and we specified a minimum length of seven amino acids. The Andromeda search engine was used for the MS/MS spectra search against the *Caenorhabditis elegans* database (downloaded from Uniprot on April 2015, containing 25,527 entries). Enzyme specificity was set as C-terminal to Arg and Lys, also allowing cleavage at proline bonds and a maximum of two missed cleavages. Dithiomethylation of cysteine was selected as fixed modification and N- terminal protein acetylation, methionine oxidation na serine/threonine/tyrosine phosphorylation as variable modifications. The “match between runs” feature of MaxQuant was used to transfer identifications to other LC-MS/MS runs based on their masses and retention time (maximum deviation 0.7 min) and this was also used in quantification experiments. Quantifications were performed with the label-free algorithms described recently. Data analysis was performed using Perseus 1.5.2.4 software (Tyanova et al., 2016).

### *In vitro* Ubiquitination Assay

HEK293T cells were transfected with pNSF-FBXO38 and lysed using an isotonic buffer containing Benzonase (0.125 U/μl). Lysates were precleared with 0.40 μm filters and Strep-tagged complexes were isolated using Strep-Tactin^®^ Sepharose resins. *In vitro* ubiquitination assays were performed as described previously (Horn et al., 2014). Briefly, resins with affinity-purified FBXO38 were mixed with 30 μl of ubiquitination assay buffer containing 0.1 μM UBE1, 10 ng/ml Ubch3, 10 ng/ml Ubch5c, 1 μM ubiquitin aldehyde (all Boston Biochem), 2.5 μg/μl ubiquitin (Sigma Aldrich), 50 mM Tris (pH 7.6), ±2 mM ATP, 5 mM MgCl2, 0.6 mM DTT for 45 min at 37°C. Complexes were eluted by competition with 3.5 μl desthiobiotin containing Buffer E (IBA Lifesciences).

### Immunocytochemistry

Cells were washed with PBS, fixed with 4% PFA in PBS for 20 min, permeabilized with 0.2% Triton X-100 in PBS for 10 min, and blocked for 1 h (3% BSA, 0.1% Triton X-100 in PBS). Incubation with indicated primary antibodies was carried out for 2 h at RT followed by incubation with Alexa Fluor-conjugated secondary antibodies (Abcam) for 30 min at RT. DAPI was used to stain DNA. To visualize F-actin, Phalloidin-iFluor 488 Reagent (Abcam) was used. Slides were mounted with ProLong Gold Antifade Mountant (Invitrogen). Images were acquired using Axio Imager Zeiss 2 (EC Plan—Nefluar objectives) and analyzed with ZEN 2.3 or ImageJ software.

### Mitotic Spreads

HCT116 cells were incubated with colchicine (5 μg/ml; Sigma Aldrich) for 90 min. Trypsinized cells were then swelled in a hypotonic solution of 75 mM KCl for 15 min at 37°C. StatSpin Cytofuge2 was used for spinning cells for 5 min at 800 rpm onto slides pre-treated with poly-L-lysine, followed by permeabilization with 0.2% Triton-X100 for 2 min and subsequent fixation with 4% paraformaldehyde for 4 min. Slides were then washed three times with PBS and blocked for 1 h (3% BSA, 0.1% Triton X-100 in PBS). Antibodies incubation was carried out as described above.

### Chromatin Immunoprecipitation

Growing cells were fixed with 1% formaldehyde for 10 min. The reaction was stopped using 125 mM glycine, cells were washed twice with PBS and collected. Afterward, the cells were lysed in a sonication buffer (50 mM Tris pH 7.5, 140 mM NaCl, 1 mM EDTA pH 8, 0.1% Triton X-100, 0.1% SDS, protease inhibitor cocktail), incubated for 10 min on ice, centrifuged and the pellets were resuspended in an identical buffer. The subsequent sonication was performed for 10 min to generate a fragment size distribution of 100–800 bp (Covaris ME220 Focused-Ultrasonicator, Peak-Power: 75 W, Duty factor: 15%, Cycle/Burst 1,000, water temperature: 6°C). The sonicated chromatin was precleared by centrifugation, diluted with an IP buffer (as described above) and immunoprecipitated with the corresponding antibodies overnight at 4°C, followed by incubation with Dynabeads-Protein G (ThermoFisher Scientific). Precipitated DNA/protein complexes were eluted from stringently washed beads using buffer containing 1% SDS and 100 mM NaHCO3. Reverse cross-linking of eluates were performed in presence of 200 mM NaCl and RnaseA (ThermoFisher Scientific; 0.15 μg/μl) overnight at 65°C, followed by 1 hour incubation with Proteinase K (Santa Cruz; 0.3 μg/μl) and subsequent purification of DNA with QIAquick PCR Purification Kit (Qiagen).

### ChIP-Seq Analysis

The quantity and quality of immunoprecipitated DNA was measured using Qubit (ThermoFisher Scientific) and analysed by Agilent 2,100 Bioanalyser (Agilent Technologies). Sequencing libraries were prepared with NEBNext Ultra II DNA (New England Biolabs) library preparation kit. Libraries were sequenced on the Illumina NextSeq 500 instrument using 76 bp single-end configuration yielding on average 27 million reads per sample.

Sequencing reads were mapped against the hg38 genome with Bowtie2 by using Galaxy Version 2.4.2 + galaxy0) with the following settings: D 20 -R 3 -N 0 -L 20 -i S,1,0.50. The resulting bam files were normalized and compared in the bamCompare utility (Galaxy), and bigwig or bam files were visualized in the UCSC genome browser. Peaks were called with MACS2 using version (2.1.1.20160309). Enriched Motifs were searched using RSAT (rsat.sb-roscoff.fr).

### RNA-Seq Analysis

Total RNA was isolated using an RNeasy Micro kit (Qiagen Sciences, Inc., Gaithersburg, Md, United States) according to the manufacturer’s protocol. The quantity and quality of isolated RNA was measured using Qubit (ThermoFisher Scientific) and analysed by Agilent 2,100 Bioanalyser (Agilent Technologies). Sequencing libraries were prepared with Takara Smarter Stranded Total RNA-seq Kit v2 Pico Input Mammalian (PN 634413). The protocol includes depletion of ribosomal RNA. Libraries were sequenced on the Illumina NextSeq^®^ 500 instrument using 76bp single-end configuration yielding on average 1.4 × 10^7^ reads per sample.

For subsequent read processing, a bioinformatic pipeline nf-core/rnaseq version 1.4.2, was used (Ewels et al., 2019). Individual steps included removing sequencing adaptors and low-quality reads with Trim Galore! (http://www.bioinformatics.babraham.ac.uk/projects/trim_galore/), mapping to reference genome GRCm38 (Ensembl annotation version 98) with HISAT2 and quantifying expression on gene level with featureCounts (Liao et al., 2014; Kim et al., 2015; Cunningham et al., 2019). Per gene mapped counts served as input for differential expression analysis using DESeq2 R Bioconductor package (Love et al., 2014). Prior to the analysis, genes not expressed in at least two samples were discarded. We supplied experimental model assuming sample group as main effect. Resulting per gene expression log2-fold changes were used for differential expression analysis. Genes exhibiting minimal absolute log2-fold change value of log2 (1.5) and statistical significance (FDR <0.05) between compared groups of samples were considered as differentially expressed. As next, gene set over representation analysis was done using gene length bias aware algorithm implemented in goseq R Bioconductor package with KEGG pathways and GO terms gene sets (Young et al., 2010).

For individual genes expression comparison, raw read counts were normalized using fragment counts per kilobase of feature length per million mapped reads (FPKM) based on sum of lengths of all gene exons.

### Microarrays

Total RNA was isolated using an RNeasy Micro kit (Qiagen Sciences, Inc., Gaithersburg, Md, United States) according to the manufacturer’s protocol. Quality and concentration of RNA were measured with a Nanodrop 2000 spectrophotometer (Thermo Fisher Scientific, Inc.). RNA integrity was analysed using an Agilent Bioanalyzer 2,100 (Agilent Technologies, Inc.). Only samples with an intact RNA profile were used for microarray analyses (RNA Integrity Number >9).

Illumina HumanHT-12 v4 Expression BeadChips (Illumina, Inc., San Diego, CA, United States) were used for the microarray analysis following the standard protocol. Briefly, 250 ng RNA was amplified with the Illumina TotalPrep RNA Amplification kit (Ambion; Thermo Fisher Scientific, Inc.) and 750 ng labelled RNA was hybridized on the chip according to the manufacturer’s protocol. Analysis was performed in indicated replicates per group. The raw data were preprocessed using GenomeStudio software (version 1.9.0.24624; Illumina, Inc.). The transcription profiles were background corrected using an Illumina proprietary correction, quantile normalized and variance stabilized using base 2 logarithmic transformation.

### Swiss Model Prediction

Models were computed by the SWISS-MODEL server homology modeling pipeline (Waterhouse et al., 2018), using ZXDB (amino acids: 361–419) and ZXDC (amino acid: 265–323) as a query. As a template Wilms’ tumor 1 (WT1) zinc finger structure (6-blw.1. A) was selected. The results of the prediction will be available in the Mendeley database.

### Quantification and Statistical Analysis

Graphs were generated using OriginPro 2021. Protein identity and similarity was assessed by MegAlign Pro (DNAStar). The mass spectrometry results are presented as a dot-plot graph with enrichment calculated as log2 differences between control and experimental samples on the *x*-axis and maximal intensity on the *y*-axis. Only proteins identified in biological replicates are indicated.

## Data Availability

The datasets presented in this study can be found in online repositories. The names of the repository/repositories and accession number(s) can be found below: ArrayExpress accession number: E-MTAB-11272, E-MTAB-11269, E-MTAB-11270.
