# Erratum: FBXO38 ubiquitin ligase controls centromere integrity *via* ZXDA/B stability

**DOI:** 10.3389/fcell.2022.1000878

**Published:** 2022-09-14

**Authors:** Frontiers Production Office

**Affiliations:** Frontiers Media SA, Lausanne, Switzerland

**Keywords:** proteasome, ubiquitin ligase, protein degradation, cullin, zinc finger protein, centromere

Due to a production error, Supplementary Table 1 was omitted from the article.

The publisher apologizes for this mistake. The original version of this article has been updated.

