# Supplementary material for: FBXO38 Ubiquitin Ligase Controls Centromere Integrity via ZXDA/B Stability
Source: Front Cell Dev Biol. 2022 Jun 23;10:929288. doi: 10.3389/fcell.2022.929288 (PMC9260856; doi:10.3389/fcell.2022.929288)
Supplement: Supplementary file 1 [file DataSheet1.pdf]

Supplementary Material

FBXO38 Ubiquitin Ligase Controls Centromere Integrity via ZXDA/B Stability

Nikol Dibus[1,2], Vladimir Korinek[3] and Lukas Cermak[1*]

* Correspondence:
Lukas Cermak, Ph.D.
lukas.cermak@img.cas.cz

Contents

Supplementary Figures ... 2
Figure S1. FBXO38 interacts with ZXDA/B in the nucleus ... 3
Figure S3. FBXO38 associates with ZXDA via zinc-finger linker motif ... 6
Figure S4. ZXDA/B do not act as transcriptional factors ... 9
Figure S6. FBXO38 controls centromeric signature via ZXDA/B stability ... 11
Supplementary table ... 12
Dataset S1. (separate file) MACS2-called peaks in ZXDA/B ChIP-seq analysis ... 12
List of reagents ... 13
Chemicals ... 13
Antibodies ... 14
Vectors ... 15
siRNAs ... 16
Oligos ... 17

Supplementary Figures

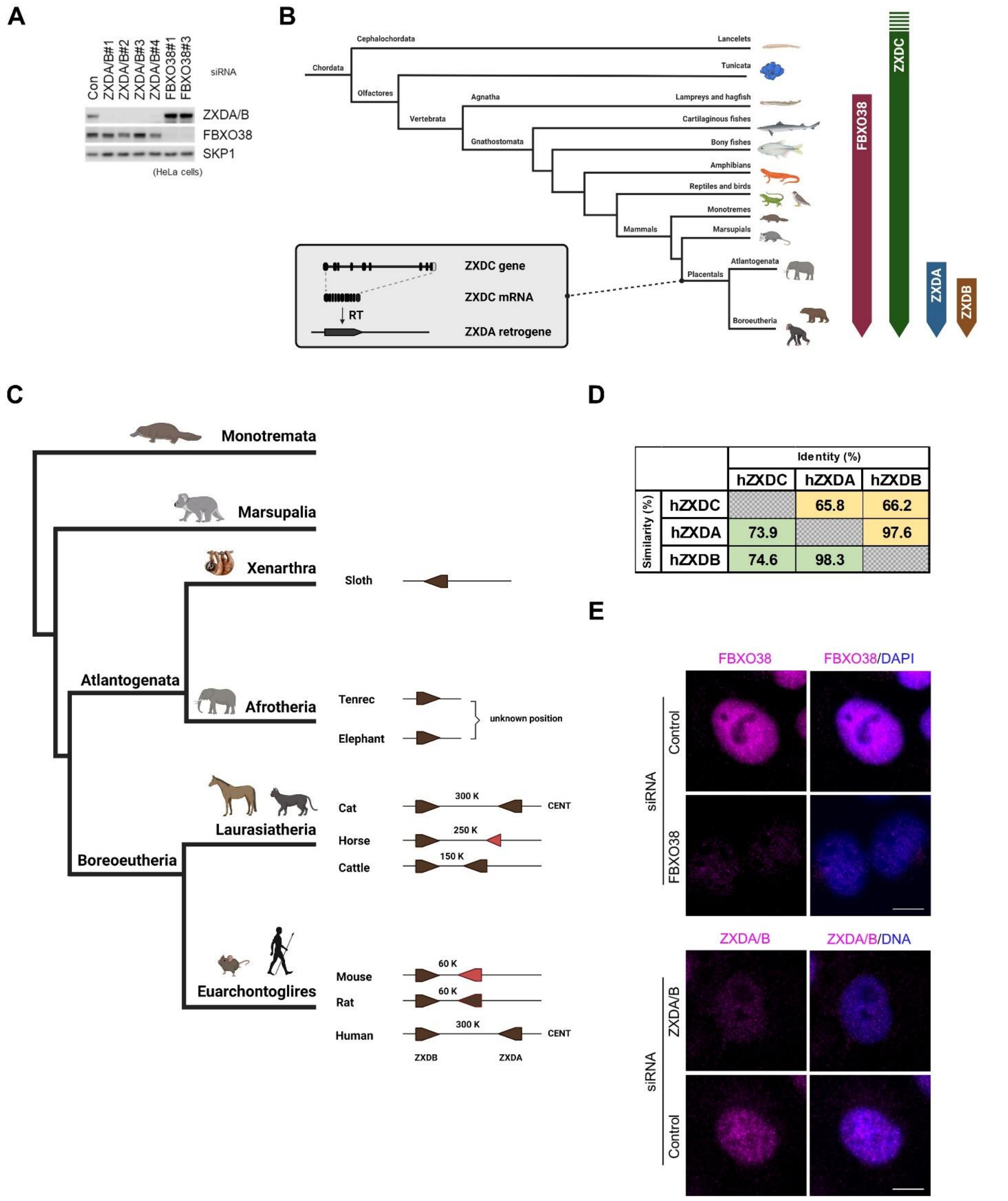

Figure S1. FBXO38 interacts with ZXDA/B in the nucleus

(A) HeLa cells were transfected with different siRNAs targeting ZXDA/B or FBXO38, and whole-cell lysates were immunoblotted as indicated. Non-targeting siRNA (Con) was used as a negative control.

(B) A simplified evolutionary scheme illustrating the emergence of *FBXO38*, *ZXDA*, *ZXDB*, *and ZXDC* genes in animals. *Zxdc* gene appeared in early animals (UniProt: Porifera - A0A1X7UJN7; Cnidaria - A7RPS8). *Zxda* and *Zxdb* genes are retrogenes that appeared in placental animals, and the *Fbxo38* gene is vertebrate-specific.

(C) A simplified evolutionary scheme illustrating the emergence of *ZXDA* and *ZXDB* genes in mammals. *ZXDA* appeared as an X-chromosome located retrogene in placental animals. In Boreoeutheria mammals, *ZXDA* duplicated and *ZXDB* appeared in an inverted position on chromosome X. In some mammals (e.g., mouse or horse), original *ZXDA* contains nonsense mutation resulting in predicted shorter protein length (in red). CENT; centromere position.

(D) Table showing the similarity and identity of human ZXD proteins calculated by MegAlign Pro.

(E) U2-OS cells were transfected with siRNA targeting FBXO38 (upper panel) or ZXDA/B (lower panel), fixed and incubated with an anti-FBXO38 (upper panel) or anti-ZXDA/B (lower panel) antibody. DNA was stained with DAPI. Scale bar, 10 µm.

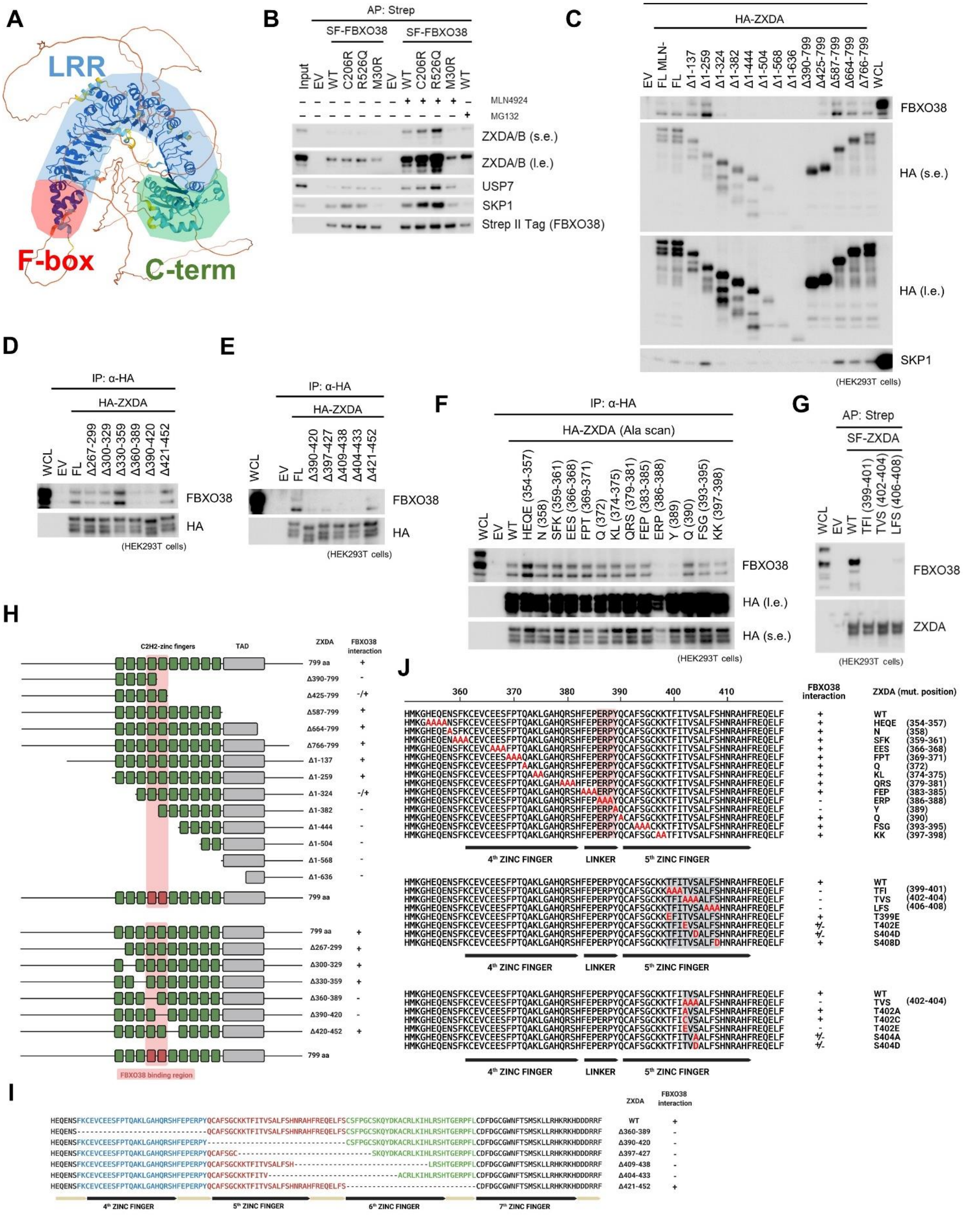
A
LRR
F-box
C-term
B
AP: Strep
SF-FBXO38
SF-FBXO38
Input
EV
WT
C206R
R526Q
M30R
EV
WT
C206R
R526Q
M30R
WT
MLN4924
MG132
ZXDA/B (s.e.)
ZXDA/B (l.e.)
USP7
SKP1
Strep II Tag (FBXO38)
C
HA-ZXDA
EV
FL MLN-
FL
Δ1-137
Δ1-259
Δ1-324
Δ1-382
Δ1-444
Δ1-504
Δ1-568
Δ1-636
Δ390-799
Δ425-799
Δ587-799
Δ664-799
Δ766-799
WCL
FBXO38
HA (s.e.)
HA (l.e.)
SKP1
(HEK293T cells)
D
IP: α-HA
HA-ZXDA
WCL
EV
FL
Δ267-299
Δ300-329
Δ330-359
Δ360-389
Δ390-420
Δ421-452
FBXO38
HA
(HEK293T cells)
E
IP: α-HA
HA-ZXDA
WCL
EV
FL
Δ390-420
Δ397-427
Δ409-438
Δ404-433
Δ421-452
FBXO38
HA
(HEK293T cells)
F
IP: α-HA
HA-ZXDA (Ala scan)
WCL
EV
WT
HEQE (354-357)
N (358)
SFK (359-361)
EES (366-368)
FPT (369-371)
Q (372)
KL (374-375)
QRS (379-381)
FEP (383-385)
ERP (386-388)
Y (389)
Q (390)
FSG (393-395)
KK (397-398)
FBXO38
HA (l.e.)
HA (s.e.)
(HEK293T cells)
G
AP: Strep
SF-ZXDA
WCL
EV
WT
TFI (399-401)
TVS (402-404)
LFS (406-408)
FBXO38
ZXDA
(HEK293T cells)
H
C2H2-zinc fingers
TAD
ZXDA
FBXO38 interaction
799 aa
Δ390-799
Δ425-799
Δ587-799
Δ664-799
Δ766-799
Δ1-137
Δ1-259
Δ1-324
Δ1-382
Δ1-444
Δ1-504
Δ1-568
Δ1-636
799 aa
799 aa
Δ267-299
Δ300-329
Δ330-359
Δ360-389
Δ390-420
Δ420-452
799 aa
FBXO38 binding region
J
FBXO38 interaction
ZXDA (mut. position)
4th ZINC FINGER
LINKER
5th ZINC FINGER
WT
HEQE (354-357)
N (358)
SFK (359-361)
EES (366-368)
FPT (369-371)
Q (372)
KL (374-375)
QRS (379-381)
FEP (383-385)
ERP (386-388)
Y (389)
Q (390)
FSG (393-395)
KK (397-398)
TFI (399-401)
TVS (402-404)
LFS (406-408)
T399E
T402E
S404D
S408D
T402A
T402C
S404A
I
ZXDA
FBXO38 interaction
WT
Δ360-389
Δ390-420
Δ397-427
Δ409-438
Δ404-433
Δ421-452
4th ZINC FINGER
5th ZINC FINGER
6th ZINC FINGER
7th ZINC FINGER

K

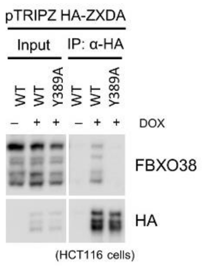

L

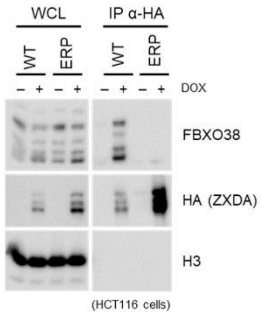

M

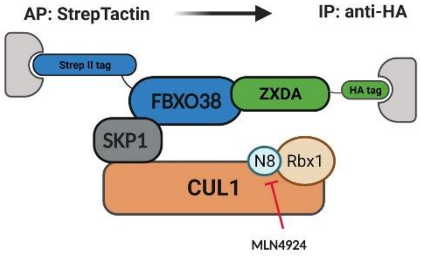

N

```
CEVCEESFPTQAKLGAHQRSHFEPERPYQCAFSGCKKTFITVSALFSHNRAHFREQELFSCSFPGCSK    hZXDA
CEVCEESFPTQAK        SHFEPERPYQCAFSGCK TFITVSALFSH       EQELFSCSFPGCSK    unmodified
                     SHFEPERPYQCAFSGCK TFITVSALFSHNR                       peptides
                                       TFITVSALFSHNR
```

	coverage (%)	peptides (N)	modified (N)	type/location	mod:unmod
ZXDA	34	33	2	pS714	2:2
FBXO38	70	91	0		
CUL1	45	56	0		
SKP1	52	12	0		

O

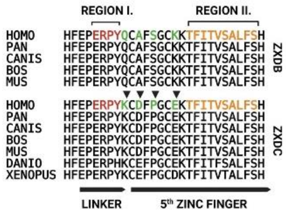

P

Protein ID	Description	Position	Sequence
79364	ZXDC, ZXDL; ZXD family zinc finger C	290..304	HFEPERPYKCDFPGC
7789	ZXDA, ZNF896; zinc finger X-linked duplicated A	382..396	HFEPERPYQCAFSGC
158586	ZXDB, ZNF905; zinc finger X-linked duplicated B	386..400	HFEPERPYQCAFSGC

Q

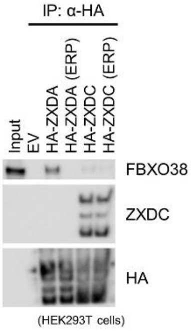

R

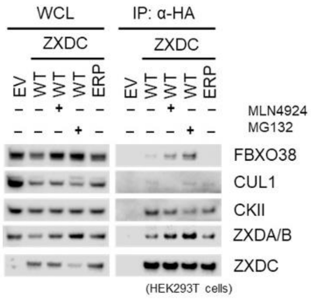

S

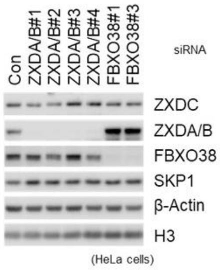

T

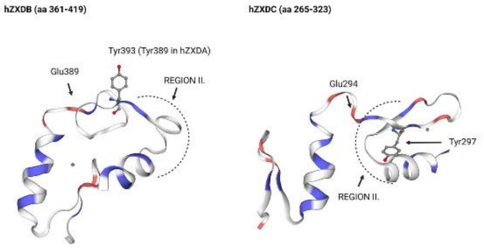

U

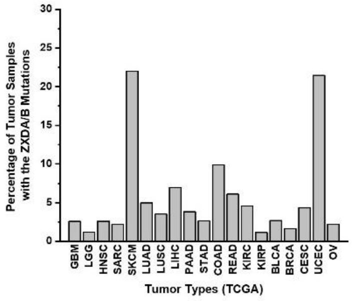

Figure S3. FBXO38 associates with ZXDA via zinc-finger linker motif

(A) Prediction of FBXO38 protein structure using AlphaFold (Jumper, Evans et al., 2021). The N-terminal part contains the F-box motif and N-terminally located leucine-rich repeats (LRRs). The C-terminal part consists of the region containing the second part of LRRs and the C-terminal domain. Mentioned parts are predicted with high accuracy (based on available AlphaFold data).

(B) HEK293T cells were transfected with StrepII-FLAG-tagged wild-type (WT) FBXO38 (SF-FBXO38 WT) or its mutated variants found in patients with motor neuronopathy (C206R; R526Q), or in twins with gender dysphoria (M30R). Where indicated, cells were treated with MLN4924 or MG-132 inhibitors for 6 hours prior to collecting. Whole-cell lysates (WCL) were subjected to affinity purification (AP) using Strep-Tactin resin and immunoblotted as indicated. Empty vector (EV) was used as a negative control; l.e. stands for long exposure, s.e. for short exposure.

(C) – (E) HEK293T cells were transfected with HA-tagged full-length (FL) ZXDA or its deletion mutants. Cells were treated with the MLN4924 inhibitor 6 hours prior to collecting. Whole-cell lysates (WCL) were subjected to immunoprecipitation (IP) using anti-HA beads. Empty vector (EV) was used as a negative control; l.e. long exposure, s.e. short exposure.

(F) HEK293T cells were transfected with HA-tagged full-length (FL) ZXDA or its mutated variants. Cells were treated, and whole-cell lysates (WCL) were subjected to immunoprecipitation as in (C). Empty vector (EV) was used as a negative control. l.e. long exposure, s.e. short exposure.

(G) HEK293T cells were transfected with SF-tagged wild-type (WT) ZXDA or its constructs with mutations of indicated amino acids. Cells were treated as in (C). Whole-cell lysates (WCL) were subjected to affinity purification (AP) using Strep-Tactin resin and immunoblotted as indicated. Empty vector (EV) was used as a negative control.

(H) Schematic representation of full-length ZXDA (1-799 aa) or its N- and C-terminally truncated variants (upper panel) or zinc finger deletion mutants (lower panel). Zinc fingers are depicted as green boxes, zinc fingers essential for interaction with FBXO38 are red-tinted. The middle panel indicates deleted amino acid positions. FBXO38-interacting variants of ZXDA are indicated by the symbol (+), (+/-) denotes reduced binding, and (-) denotes a lack of binding. TAD; transcriptional activation domain.

(I) Detail of the sequence surrounding the 4th-7th zinc fingers of ZXDA protein and their deletions used in the study (S3E). Deleted zinc fingers and the following linkers are color-coded. The lower line shows the position of the zinc fingers (black) and linkers (ocher). The middle panel indicates deleted amino acid positions. Mutants interacting with FBXO38 are designated with (+) in the right part of the panel.

(J) Schematic representation of ZXDA mutants. ZXDA mutants found to interact with FBXO38 are indicated by the symbol (+). (+/-) denotes reduced binding, and (-) denotes a lack of binding. Positions and amino acids mutated are depicted in the right panel.

(K) – (L) HCT116 cells with inducible expression of HA-tagged wild-type (WT) ZXDA or its mutants were treated with doxycycline where indicated. Whole-cell lysates (WCL) were subjected to immunoprecipitation (IP) using anti-HA beads.

(M) Schematic representation of the tandem affinity purification strategy and proteomic analysis of ZXDA modifications encompassing the FBXO38-interacting region. SF-tagged FBXO38 and HA-tagged ZXDA were co-expressed in HEK293T cells. The cells were treated with the MLN4924 inhibitor 6 hours prior to collecting and subjected to isotonic lysis and affinity purification (AP) using Strep-Tactin resin followed by elution with desthiobiotin and subsequent immunoprecipitation (IP) using anti-HA magnetic beads. The composition and modifications of

purified peptides were analyzed using Liquid Chromatography with Tandem Mass-Spectrometry (LC-MS/MS).

(N) Identified peptides (from S3M) covering the FBXO38-interacting ZXDA region (upper panel). The table (lower panel) summarizes the LC-MS/MS analysis results showing the coverage of sequences, the total number of identified peptides, and their modifications. The rightmost columns indicate the identified phosphorylation of ZXDA in position S714 and the ratio between modified and unmodified peptides identified in tandem purification of FBXO38-ZXDA complex.

(O) Alignment of ZXDA/B and ZXDC sequences encompassing the region of the linker and the 5th zinc finger. The regions indicated above represent essential motifs involved in ZXDA/B interaction with FBXO38 – zinc finger linker (Region I.) and amino acids located in 5th zinc finger (Region II.). Arrowheads point to amino acids that differ between ZXDA/B and ZXDC. Amino acids encompassing this region are depicted below, with a zinc-finger linker necessary for FBXO38-interaction highlighted in red (degron). Mutations of amino acids marked in yellow had a negative effect on FBXO38-interaction. The green-labeled amino acids differ between ZXDA/B and ZXDC.

(P) Analysis of degenerate motif H-X(3)-[ED]-[RK]-P-[YF]-x-C-x(3,5)-C occurrence using MOTIF Search (https://www.genome.jp/tools/motif/MOTIF2.html) and human Kyoto Encyclopedia of Genes and Genomes database (KEGG). Specific sequences and their positions are indicated on the right side of the table.

(Q) HEK293T cells were transfected with HA-tagged ZXDA or ZXDC or their ERP mutants. Cells were treated with the MLN4924 inhibitor 6 hours prior to collecting. Whole-cell lysates (WCL) were subjected to immunoprecipitation (IP) using anti-HA beads. Empty vector (EV) was used as a negative control.

(R) HEK293T cells were transfected with HA-tagged ZXDC or its ERP mutant. Where indicated, cells were treated with either MG-132 or the MLN4924 inhibitor 6 hours prior to collecting. Whole-cell lysates (WCL) were subjected to immunoprecipitation (IP) using anti-HA beads. Empty vector (EV) was used as a negative control.

(S) HeLa cells were transfected with different siRNAs targeting ZXDB or FBXO38, and whole-cell lysates were immunoblotted as indicated. Non-targeting siRNA (Con) was used as a negative control. The blot is the same used for the siRNA efficiency test in Figure (S1E).

(T) The protein structure prediction of hZXDB and hZXDC 4th and 5th zinc fingers. SWISS MODEL-based (https://swissmodel.expasy.org/) homology modeling was used to predict the structure of the zinc-finger region containing FBXO38-dependent degron. hZXDB with essential glutamate (E389), tyrosine (Y393), and the fifth zinc finger region highlighted are shown (left). The prediction of the homologous region in hZXDC with identical amino acids and regions highlighted (right).

(U) Frequency of tumor samples with ZXDA/B mutations identified in The Cancer Genome Atlas (TCGA) cohort. Single abbreviations represent different groups of cancers (e.g., Skin Cutaneous Melanoma – SKCM). The results are based upon data generated by the TCGA Research Network (https://www.cancer.gov/tcga).

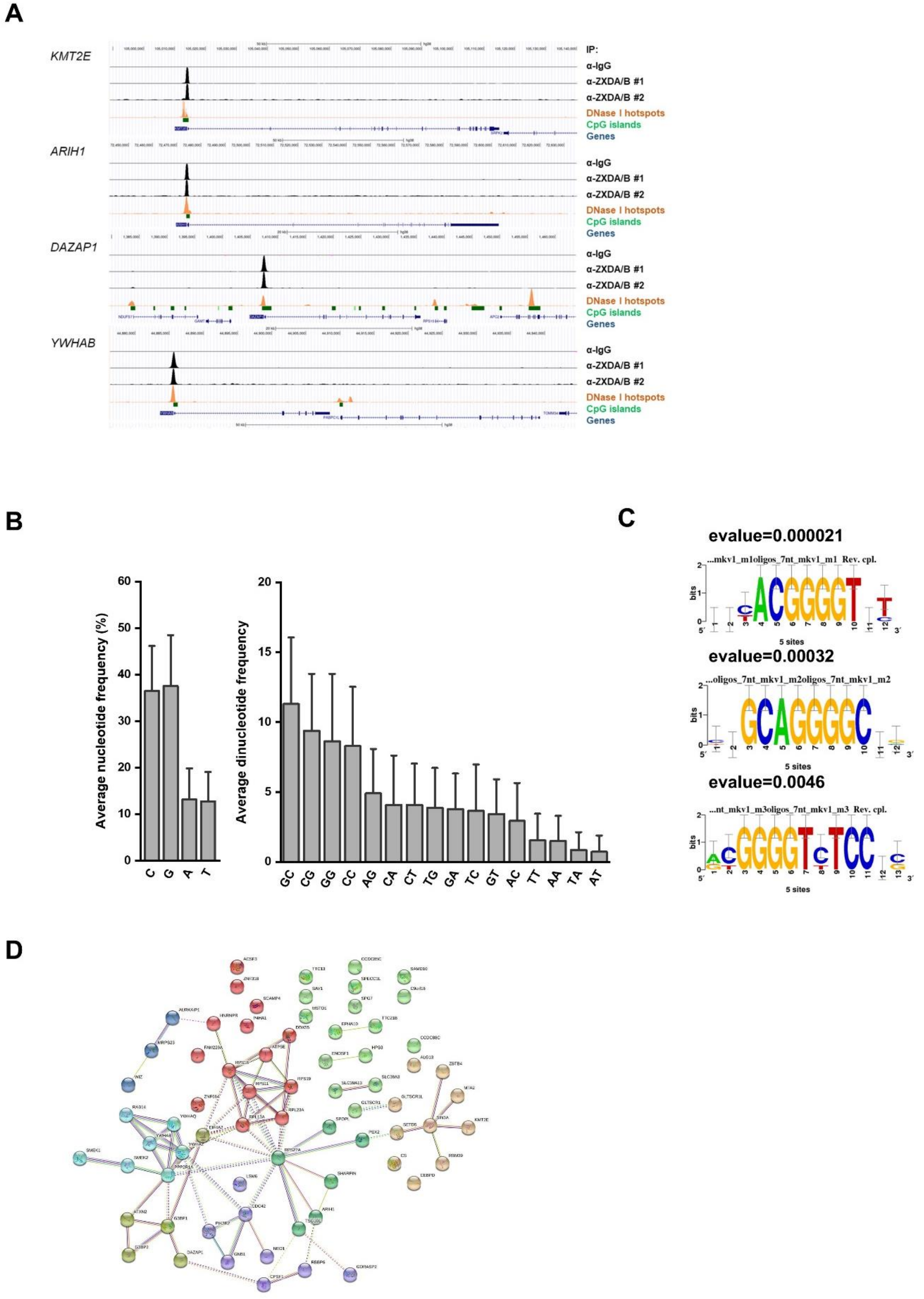
A
KMT2E
ARIH1
DAZAP1
YWHAB
IP:
α-IgG
α-ZXDA/B #1
α-ZXDA/B #2
DNase I hotspots
CpG islands
Genes
B
Average nucleotide frequency (%)
C G A T
Average dinucleotide frequency
GC CG GG CC AG CA CT TG GA TC GT AC TT AA TA AT
C
evalue=0.000021
...mkv1_m1oligos_7nt_mkv1_m1 Rev. cpl.
5 sites
evalue=0.00032
...oligos_7nt_mkv1_m2oligos_7nt_mkv1_m2
5 sites
evalue=0.0046
...nt_mkv1_m3oligos_7nt_mkv1_m3 Rev. cpl.
5 sites
D

Figure S4. ZXDA/B do not act as transcriptional factors

(A) Selected ChIP-seq tag profiles of two independent ZXDB ChIP-seq samples. Five peaks selected as significant called by the MACS2 program are shown. Lower panels show DNase I hypersensitivity regions (orange), CpG islands (green), and exon-intron structure of genes (blue). The scheme was generated using UCSC Genome Browser (Kent, Sugnet et al., 2002).

(B) Nucleotide composition of ZXDB-precipitated chromatin peaks. Twenty base pairs surrounding peaks called by MACS2 were analyzed using RSAT software for nucleotide and motifs enrichment.

(C) Significant cis-motifs identified by RSAT oligo-analyzer motif discovery tool. Three motifs with the lowest expectation (E)-values and high log-likelihood ratio are shown. The X- and Y-axes show the position of nucleotides and the bits score, respectively.

(D) Protein/genes interaction map. Only genes with promoters significantly enriched in ZXDB ChIP samples were analyzed. The map was constructed using the STRING 11.5 web tool (Szklarczyk, Gable et al., 2019). Colored lines denote interactions: green (text mining), blue (curated database), purple (experimentally determined), and black (co-expression).

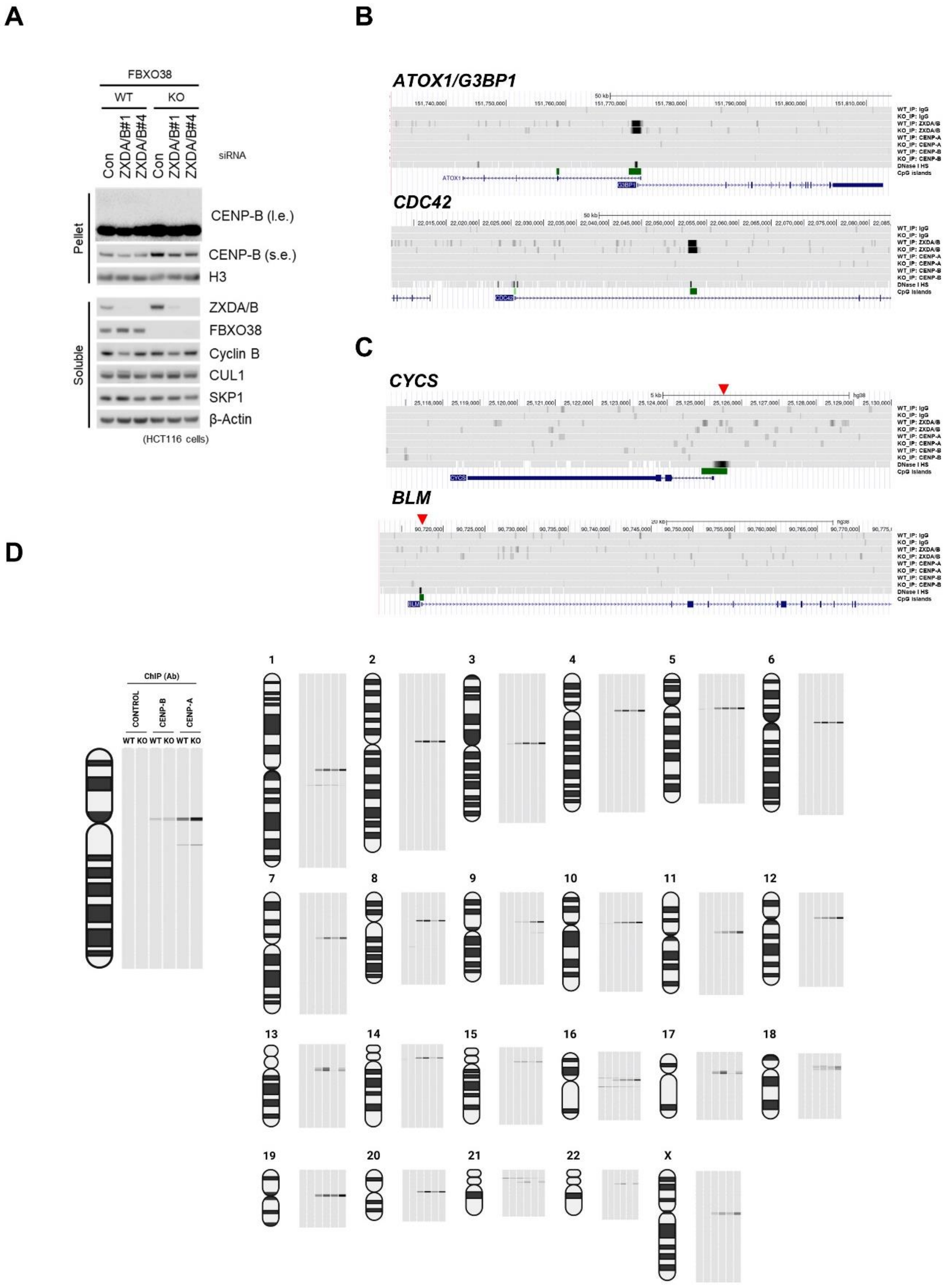
A
FBXO38
WT
KO
Con
ZXDA/B#1
ZXDA/B#4
Con
ZXDA/B#1
ZXDA/B#4
siRNA
Pellet
CENP-B (l.e.)
CENP-B (s.e.)
H3
Soluble
ZXDA/B
FBXO38
Cyclin B
CUL1
SKP1
β-Actin
(HCT116 cells)
B
ATOX1/G3BP1
CDC42
C
CYCS
BLM
WT_IP: IgG
KO_IP: IgG
WT_IP: ZXDA/B
KO_IP: ZXDA/B
WT_IP: CENP-A
KO_IP: CENP-A
WT_IP: CENP-B
KO_IP: CENP-B
DNase I HS
CpG Islands
D
ChIP (Ab)
CONTROL
CENP-B
CENP-A
WT KO WT KO WT KO
1
2
3
4
5
6
7
8
9
10
11
12
13
14
15
16
17
18
19
20
21
22
X

Figure S6. FBXO38 controls centromeric signature via ZXDA/B stability

(A) FBXO38 wild type (WT) and knockout (KO) HCT116 cells were transfected with two different siRNAs targeting ZXDA/B, or with control siRNA. The cells were subjected to isotonic lysis 48 hours after transfection, soluble fractions were collected and the pellets were further lysed in the presence of Benzonase nuclease and subsequently heat-denatured in the presence of 1% SDS for insoluble fractions. Lysates were immunoblotted as indicated. This image represents the longer exposure of CENP-B immunostaining of the blot found in (Figure 6C).

(B) Examples of ChIP-Seq analysis in FBXO38 WT and KO HCT116 cell line. ZXDA/B-associated *G3BP1* (promoter localized) and *CDC42* (intron localized) loci were selected. Fragmented chromatin was immunopurified with either non-specific IgG, or ZXDA/B, CENP-A, and CENP-B polyclonal antibodies. Trimmed reads were aligned using the Bowtie2 program and further normalized with bamCoverage utility (Galaxy – DeepTools) to obtain counts per million (CPM) values. Coverage of the human genome (hg38) was visualized in the UCSC genome browser (Kent et al., 2002). The quantity of precipitated chromatin is shown as a density graph using autoscale to highlight the enrichment. The upper two lines represent the control profiles, followed by anti-ZXDA/B, anti-CENP-A, and anti-CENP-B ChIP-seq samples. For each antibody, data from FBXO38 WT and KO cell lines are present. Lower panels show the location of genes (blue), DNase I hypersensitivity regions, and CpG islands.

(C) The quantity of precipitated chromatin is shown as a density graph of *CYCS* and *BLM* loci using autoscale to highlight the enrichment. Red arrowheads indicate previously described CENP-A depositions at the DNase I hypersensitive sites. Data are generated and visualized as in (B).

(D) The quantity of precipitated chromatin is shown as a density graph using autoscale to highlight the centromeric enrichment. All human chromosomes (except Y) are present. The single lines next to the chromosome schemes represent ChIP results using IgG control, CENP-B, and CENP-A antibodies (in FBXO38 WT and KO HCT116 cells). Data are generated and visualized as in (B).

Supplementary table

Dataset S1. (separate file) MACS2-called peaks in ZXDA/B ChIP-seq analysis

MACS2-called ZXDA-associated annotated peaks (version 2.1.1.20160309). Q-values were calculated from p-values using Benjamini-Hochberg procedure (--qvalue). Galaxy MACS2 callpeak app was used for calling peaks and calculating q-values. Peaks were annotated by PAVIS online software (https://manticore.niehs.nih.gov/pavis2/annotate).

List of reagents

Chemicals

Name	Company	CAT#
2-mercaptoethanol	Sigma	M6250
4',6-diamidin-2-fenylindol (DAPI)	Sigma	D9542
ATP	Sigma	A26209
Benzonase	Santa Cruz	sc-391121
Blasticidin	Sigma	15205
Colchicine	Sigma	C3915
Cycloheximide	Sigma	C7698
Doxycycline hyclate	Sigma	D9891
Dithiothreitol	Sigma	10197777001
Formaldehyde	Thermo Fisher Scientific	28908
Lipofectamine 2000	Thermo Fisher Scientific	11668019
Methanol	Penta	67-56-1
MG132	Medchemexpresss	HY-13259
MLN4924	Medchemexpresss	HY-70062
Sodium fluoride	Sigma	S7920
Sodium thiocyanate	Sigma	251410
Sodium orthovanadate	Sigma	450243
Paraformaldehyde	Electron Microscopy Sciences	15710
Polybrene	Sigma	107689
Polyethyleneimine (MW 25 000)	Polysciences	23966
Protease Inhibitors Mini Tablets	Pierce	A32955
Proteinase K	Sigma	P2308
Puromycin	Sigma	P8833
RNAiMax	Thermo Fisher Scientific	13778150
Sodium dodecyl sulfate (SDS)	Sigma	71736
UBE1	Boston Biochem/R&D Systems	E-305-025
UBCH3	Boston Biochem/R&D Systems	E2-610-100
Ubch5c	Boston Biochem/R&D Systems	E2-627-100
ubiquitin aldehyde	Boston Biochem/R&D Systems	U-201-050
Ubiquitin	Boston Biochem/R&D Systems	U-100H-10M

Antibodies

Primary antibodies	Source	CAT#	RRID	LINK
Anti-centromere antibodies	Antibodies Incorporated	15-234		
ARP3	Santa Cruz	ab49671	AB_2257830	AB_2257830
BAZ1A	Santa Cruz	sc-393164		
CDH1	Thermo Fisher Scientific	34-2000	AB_2533160	AB_2533160
CENP-A	Cell Signaling	2186	AB_10828491	AB_10828491
CENP-B	Santa Cruz	sc-376283	AB_10988421	AB_10988421
CENP-B	Santa Cruz	sc-376392	AB_11151020	AB_11151020
CENP-B	Atlas Antibodies	HPA054405	AB_2682477	AB_2682477
CENP-C	Atlas Antibodies	HPA058252	AB_2683654	AB_2683654
CK-IIα	Santa Cruz	sc-514403	AB_2894823	AB_2894823
CKII-β	Santa Cruz	sc-12739	AB_626792	AB_626792
CP110	Bethyl	A301-343A	AB_937760	AB_937760
CUL1	Thermo Fisher Scientific	32-2400	AB_2533070	AB_2533070
Cyclin B1	Santa Cruz	sc-245	AB_627338	AB_627338
DDB1	Santa Cruz	sc-376860	AB_2894825	AB_2894825
FBXO11	Novus Biologicals	NB100-59825	AB_892468	AB_892468
FBXO28	Bethyl	A302-377A	AB_1907260	AB_1907260
FBXO38	Atlas Antibodies	HPA041444	AB_2677484	AB_2677484
FLAG	Cell Signaling	14793	AB_2572291	AB_2572291
GFP	Cell Signaling	2956	AB_1196615	AB_1196615
H3	Abcam	ab1791	AB_302613	AB_302613
HA	Cell Signaling	3724	AB_1549585	AB_1549585
Lamin B	Santa Cruz	sc-6216	AB_648156	AB_648156
p21	Santa Cruz	sc-397	AB_632126	AB_632126
p27	Santa Cruz	sc-528	AB_632129	AB_632129
p53	Cell Signaling	2527	AB_10695803	AB_10695803
PARP-1	Santa Cruz	sc-8007	AB_628105	AB_628105
Phospho-β-Catenin (Ser33/37)	Cell Signaling	2009	AB_2088238	AB_2088238
REST	Millipore	07-579	AB_11211936	AB_11211936
RRM2	Santa Cruz	sc-398294	AB_2894824	AB_2894824
SKP1	Cell Signaling	12248	AB_2754993	AB_2754993
SKP2	Thermo Fisher Scientific	32-3300	AB_2533074	AB_2533074
Strep II Tag	Novus Biologicals	NBP2-43735		
StrepTactin HRP	Bio-Rad	1610381		
USP7	Bethyl	A303-943A	AB_2620292	AB_2620292
ZXDA/B	Bethyl	A303-656A	AB_11205808	AB_11205808
ZXDA/B	Atlas Antibodies	HPA043789	AB_2678673	AB_2678673
α-Tubulin	Proteintech	66031-1-Ig	AB_11042766	AB_11042766
β-Actin	Santa Cruz	sc-69879	AB_1119529	AB_1119529
β-TrCP2	Cell Signaling	4394	AB_10545763	AB_10545763

Secondary antibodies	Source	CAT#	RRID	LINK
Anti-Rabbit IgG (Alexa Fluor® 647)	Abcam	ab150067	AB_2894821	AB_2894821
Anti-Rabbit IgG (Alexa Fluor® 555)	Abcam	ab150070	AB_2783636	AB_2783636
Anti-Human IgG (Alexa Fluor 488)	Thermo Fisher Scientific	A11013	AB_2534080	AB_2534080
Anti-Mouse IgG (Alexa Fluor 647)	Abcam	ab150115	AB_2687948	AB_2687948

Fluorescence probes	Source	CAT#
Phalloidin - Fluor 488	Abcam	ab176753

Beads for protein purification	Source	CAT#
anti-HA (magnetic)	Pierce	88837
Strep-Tactin® Superflow resin	IBA	2-1206-025
Protein G (magnetic)	Dynabeads	10004D
ChromoTek GFP-Trap® (magnetic)	Chromotek	gtma-20

Vectors

Expression vectors	Backbone	N-term. tag
ZXDA	pCDNA3	2xStrep-1xFLAG
ZXDA	pCDNA3	1xHA
ZXDA	pSB	2xStrep-1xFLAG
ZXDA	pTRIPZ	1xHA
ZXDC	pCDNA3	2xStrep-1xFLAG
FBXO38	pCDNA3	2xStrep-1xFLAG
FBXO38	pSB	2xStrep-1xFLAG
DEDD2	pCDNA3	2xStrep-1xFLAG
CUL1	pCDNA3	1xHA
CENP-B	pKG141	GFP

Sleeping Beauty System	Company	Cat. Number
pSBtet-Pur	Addgene	60507
pSB100X	Addgene	34879

Lentiviral packaging vectors	Company	Cat. Number
pLenti CMV/TO SV40 Small and Large T antigen	Addgene	22298
pCMV-dR8.2	Addgene	8455
pCMV-VSV-G	Addgene	8454

CRISPR system	Company	Insert	Targeted cells
pXPR001	Addgene	FBXO38_5' #1	HCT116
pXPR001	Addgene	FBXO38_5' #2	RPE-1
pXPR001	Addgene	FBXO38_3' #1	HCT116/RPE-1

siRNAs

Name	Sequence	Target	Position
FBXO38#1	GGGUGUAUUUCAGCGAGUAUU	FBXO38	TR
FBXO38#2	GGACUCGAUUGGUUGAUAUUU	FBXO38	TR
FBXO38#3	GAGCGAAGCUGUUUGAGUAUU	FBXO38	UTR
ZXDA/B (#1)	GCUCUGUGGUGUUGGAUAAUU	ZXDB*	UTR
ZXDA/B (#2)	CUGAAAGGCCACAGCAUAAUU	ZXDA/B/C	TR
ZXDA/B (#3)	CCAAGAAGCACCAGCUGAAUU	ZXDA/B/C	TR
ZXDA/B (#4)	ACACAUAAGUCUUGGAAAUUU	ZXDB*	UTR

*Oligos were used in HeLa cells (Figure S1A) and HCT116 (Figure S6A) that express only ZXDB.

Oligonucleotides

Cloning oligonucleotides

Gene	Orientation	Position	Sequence	RE SITE
FBXO38	FWD	FL	CGAGAAAGGAGCTAGCGGGCCACGAAAGAAAAGTG	NHE1
FBXO38	REV	FL	GGGGTCGACTTAAATGTAGTCATCTTCAA	SAL1
X38_1090_REV	REV	1090	GGGGTCGACTCAAAATACACCCTTCTTCATCTG	SAL1
CUL1	FWD	FL	GGGGATCCTCGTCAACCCGGAGCCAGAA	BAMH1
CUL1	REV	FL	GGGGCGGCCGCTTAAGCCAAGTAACTGTAGGTGT	NOT1
DEDD2	FWD	FL	GGGGATCCGCGCTATCCGGGTCGAC	BAMH1
DEDD2	REV	FL	GGCTCGAGTCAGGAGGCCTCTGTCGGG	XHO1
ZXDA	FWD	FL	GGGGATCCGAAATCCCGAAGCTGCTCCC	BAMH1
ZXDA	REV	FL	GGCTCGAGTCATACCAAAAATGAGCTGCCAG	XHO1
ZXDA	FWD	138	GGGGATCCCAGGGCCCCCACTGCCTGTC	BAMH1
ZXDA	FWD	260	GGGGATCCTCTGGTCCAGGCGTGGTGCT	BAMH1
ZXDA	FWD	325	GGGGATCCGATAAACTGCGGCCCTTCGG	BAMH1
ZXDA	FWD	383	GGGGATCCTTCGAACCCGAGAGGCCTTA	BAMH1
ZXDA	FWD	445	GGGGATCCACCGGCGAGAGACCTTTCCT	BAMH1
ZXDA	FWD	505	GGGGATCCCTGGGCACAAAGCCTTTCGT	BAMH1
ZXDA	FWD	569	GGGGATCCAAGGTGGGCCAGGATCTCTT	BAMH1
ZXDA	FWD	637	GGGGATCCAGCTCGACTCTGGCAGGGCA	BAMH1
ZXDA	REV	389	GGCTCGAGTCAGTAAGGCCTCTCGGGTTCGA	XHO1
ZXDA	REV	424	GGCTCGAGTTAAAACAGTTCCTGTTCCCTGAAAT	XHO1
ZXDA	REV	586	GGCTCGAGTCAGGGTGTAAGAGAATTTGCTGC	XHO1
ZXDA	REV	663	GGCTCGAGTCAGCCATCAAGGACGGAGGGTC	XHO1
ZXDA	REV	765	GGCTCGAGTCATGGTGTCAGTAGTTCTGAGA	XHO1
ZXDC	FWD	FL	GGGGATCCGACCTCCCGGCGCTGCTCCC	BAMH1
ZXDC	REV	FL	GGCTCGAGTCACTGCAGATCCTGCAGGTTGA	XHO1

Mutagenesis oligonucleotides

Gene	Mutation	Sequence
FBXO38	M30A	CAGCAGATGAAACAAAGGACTATAGGAATCAACTTTCACATGAAG
FBXO38	C206R	TGCCTTAGCATTGGGATACGAGGAATTTCAGGAACATTC
FBXO38	R526Q	CGAGGAGAATGACTTTCAGCAAGATCTGCAGCCAG
FBXO38	Δ30-65 (ΔFBOX)	TCTCACAACTCGCAGATATAGATAGTCCTTTGTTTCATCTGC
ZXDA	HEQE354-357AAAA	CTCAAGGCGCACATGAAGGGCGCGGCCGCCGCGAACTCGTTCAAATGTGAGGT
ZXDA	N358A	GAAGGGCCATGAGCAGGAGGCCTCGTTCAAATGTGAGGTG
ZXDA	SFK359-361AAA	GGGCCATGAGCAGGAGAACGCGGCCGCCTGTGAGGTGTGCGAGGAG
ZXDA	EES366-368AAA	TCAAATGTGAGGTGTGCGCGGCCGCCTTCCCCACGCAGGCCAA
ZXDA	FPT369-371AAA	GTGTGCGAGGAGAGCGCGGCCGCCCAGGCCAAACTCG
ZXDA	Q372A	GAGAGCTTCCCCACGGCGGCCAAACTCGGCGC
ZXDA	AKL373-5AAA	GCTTCCCCACGCAGGCGGCCGCCGGCGCCCACCAGCGC
ZXDA	QRS379-381AAA	GCCAAACTCGGCGCCCACGCGGCCGCCCACTTCGAACCCGAGAG
ZXDA	FEP383-5AAA	CACCAGCGCAGCCACGCGGCCGCCGAGAGGCCTTACC
ZXDA	ERP386-388AAA	AGCCACTTCGAACCCGCGGCCGCTTACCAGTGCGCGTT
ZXDA	Y389A	AAAACGCGCACTGGGCAGGCCTCTCGGGTTCG
ZXDA	Y389F	ACGCGCACTGGAAAGGCCTCTCGGG
ZXDA	Q390A	AACCCGAGAGGCCTTACGCGTGCGCGTTTTCTGGCT
ZXDA	FSG393-395AAA	AGAGGCCTTACCAGTGCGCGGCCGCTGCCTGCAAGAAGACATTTATCAC
ZXDA	TFI399-401AAA	GTGCGCGTTTTCTGGCTGCAAGAAGGCGGCCGCCACAGTGAGTGCTCTGTTTT CCCAT
ZXDA	TVS402-404AAA	GCGTTTTCTGGCTGCAAGAAGACATTTATCGCGGCCGCTGCTCTGTTTTCCCAT AACCGC
ZXDA	LFS406-408AAA	TGCAAGAAGACATTTATCACAGTGAGTGCGGCCGCTGCCCATAACCGCGCCC ATTTCAGG
ZXDA	KK397-398AA	ACCAGTGCGCGTTTTCTGGCTGCGCGGCGACATTTATCACAGTGAGTGCTC
ZXDA	Δ266-330	AGGCGTGGTGCTGTTCGGCTGCCCTG
ZXDA	Δ267-299	CCAGGCGTGGTGCTGTTCAAATGCCCCCTG
ZXDA	Δ299-360	ACACCTCACATTTGAAGGGCCTCTGGCCCTG
ZXDA	Δ300-329	AGGGCAGCCGAAGGGCCTCTGGCC
ZXDA	Δ329-389	CGATAAACTGCGGCCCTACCAGTGCGCGTTTT
ZXDA	Δ330-359	ACGATAAACTGCGGCCCTTCAAATGTGAGGTGTG
ZXDA	Δ360-389	TGAGCAGGAGAACTCGCAGTGCGCGTTTTCTG
ZXDA	Δ397-427	CCTTGTCATATTGCTTGCTGCAGCCAGAAAACGCGCAC
ZXDA	Δ404-433	CAGGTGAATTTTCAGCCTACAAGCCACTGTGATAAATGTCTTCTTGCA
ZXDA	Δ409-438	GTGACTCCGCAGGTGAATGGAAAACAGAGCACTCAC
ZXDA	Δ421-452	AGCCACAGCCATCAAAGTCGCAGGAAAACAGTTCCTGT
ZXDC	ERP294-296AAA	GCGCAGCCACTTCGAGCCCGCGGCCGCTTACAAGTGTGACTTTCC

CRISPR cloning oligonucleotides

Name	Orientation	Sequence
FBXO38_5' #1	FWD	CACCGAAGCTGTATGACCGTATGTG
FBXO38_5' #1	REV	AAACCACATACGGTCATACAGCTTC
FBXO38_5' #2	FWD	CACCGAGAAACTCGTATTTATCAAG
FBXO38_5' #2	REV	AAACCTTGATAAATACGAGTTTCTC
FBXO38_3' #1	FWD	CACCGGATGCATGTTTTCCGGTGAA
FBXO38_3' #1	REV	AAACTTCACCGGAAAACATGCATCC

CRISPR verification oligonucleotides

Name	Orientation	Sequence
FBXO38	FWD	CCTGTGGAGGGAGCTCAATA
FBXO38	REV	GGTGCAGTTTAGTTGCAGAGG
